# The pomegranate (*Punica granatum* L.) draft genome dissects genetic divergence between soft‐ and hard‐seeded cultivars

**DOI:** 10.1111/pbi.13260

**Published:** 2019-11-06

**Authors:** Xiang Luo, Haoxian Li, Zhikun Wu, Wen Yao, Peng Zhao, Da Cao, Haiyan Yu, Kaidi Li, Krishna Poudel, Diguang Zhao, Fuhong Zhang, Xiaocong Xia, Lina Chen, Qi Wang, Dan Jing, Shangyin Cao

**Affiliations:** ^1^ Chinese Academy of Agricultural Sciences Zhengzhou Fruit Tree Research Institute Zhengzhou China; ^2^ State Key Laboratory of Ophthalmology Zhongshan Ophthalmic Center Sun Yat‐sen University Guangzhou China; ^3^ National Key Laboratory of Wheat and Maize Crop Science College of Life Sciences Henan Agricultural University Zhengzhou China; ^4^ Key Laboratory of Resource Biology and Biotechnology in Western China Ministry of Education College of Life Sciences Northwest University Xi'an China; ^5^ School of Biological Sciences University of Queensland Brisbane Qld Australia; ^6^ Biomarker Technologies Corporation Beijing China

**Keywords:** genome, single‐molecule technologies, divergence, seed, *Punica granatum* L

## Abstract

Complete and highly accurate reference genomes and gene annotations are indispensable for basic biological research and trait improvement of woody tree species. In this study, we integrated single‐molecule sequencing and high‐throughput chromosome conformation capture techniques to produce a high‐quality and long‐range contiguity chromosome‐scale genome assembly of the soft‐seeded pomegranate cultivar ‘Tunisia’. The genome covers 320.31 Mb (scaffold N50 = 39.96 Mb; contig N50 = 4.49 Mb) and includes 33 594 protein‐coding genes. We also resequenced 26 pomegranate varieties that varied regarding seed hardness. Comparative genomic analyses revealed many genetic differences between soft‐ and hard‐seeded pomegranate varieties. A set of selective loci containing *SUC8‐like*,*SUC6*,* FoxO* and *MAPK* were identified by the selective sweep analysis between hard‐ and soft‐seeded populations. An exceptionally large selective region (26.2 Mb) was identified on chromosome 1. Our assembled pomegranate genome is more complete than other currently available genome assemblies. Our results indicate that genomic variations and selective genes may have contributed to the genetic divergence between soft‐ and hard‐seeded pomegranate varieties.

## Introduction

Pomegranate (*Punica granatum* L.) is an important edible fruit tree species native to central Asia. The fruit has gained widespread popularity because of its functional and nutraceutical properties (Johanningsmeier and Harris, 2011). The properties of pomegranate juices, seeds and extracts are potentially beneficial for treating cardiovascular disease, diabetes and prostate cancer (Patel *et al*., 2008). Traditional hard‐seeded pomegranate varieties are well adapted to the cold environment, whereas the soft‐seeded varieties have become popular with consumers as their fruits are easily swallowed. Elucidating the phenotypic variations between soft‐ and hard‐seeded pomegranate varieties is crucial for the molecular marker‐assisted selection breeding of new soft‐seeded cultivars in breeding programmes.

Complex geographical interactions, natural selection, hybridization and domestication pressures have likely contributed to the phenotypic divergence between soft‐ and hard‐seeded pomegranate varieties (Cappellini *et al*., 2018; Martín‐Robles *et al*., 2017; Sylvain and Thomas, 2010), which can likely be decoded by comparing genomes and analysing population genetic divergence. ‘Taishanhong’ and ‘Dabenzi’ are two representative hard‐seeded pomegranate cultivars in China. The current ‘Dabenzi’ pomegranate genome assembly, which is based on short‐read sequencing, was first published by Qin *et al*. (2017). This assembly indicates that a Myrtales lineage‐specific whole‐genome duplication event occurred in the common ancestor before the divergence of pomegranate and eucalyptus. Yuan *et al*. (2017) subsequently released the ‘Taishanhong’ pomegranate genome, which resolved the previously debated taxonomic status of the genus *Punica,* and reclassified it in the family Lythraceae. These two genomes enabled certain advances in comparative and evolutionary genomics studies (Jia *et al*., 2018; Qin *et al*., 2017; Yuan *et al*., 2017; Zhao *et al*., 2018). However, these resources are fragmented, and almost of all of the scaffolds or contigs are arbitrarily ordered and oriented. This greatly impedes map‐based cloning studies and hampers the identification of crucial intergenic regions related to valuable traits, including the divergence between soft‐ and hard‐seeded pomegranate varieties.

Previous studies on the divergence between soft‐ and hard‐seeded pomegranate varieties have focused on seed hardness. For example, gene expression analyses revealed that lignin and cellulose biosynthetic genes, including *CCR*,* CAD*,* CelSy*,* SuSy*,* CCoA‐OMT*,* MYB*,* WRKY* and *MYC*, are differentially expressed between soft‐ and hard‐seeded pomegranate varieties at different growth stages (e.g. fruit setting to ripening stages) (Xue *et al*., 2017; Zarei *et al*., 2016). Quantitative proteomics and microRNA sequencing results have suggested that genes altering the cell wall structure contribute to the differences in seed hardness between soft‐ and hard‐seeded pomegranates (Luo *et al*., 2018; Niu *et al*., 2018). Four seed hardness‐related quantitative trait loci, potentially explaining 15% to 30% of the observed phenotypic variation, were identified by Harel‐Beja *et al*. (2015). However, considerable work is still required before robust molecular markers related to the divergence between soft‐ and hard‐seeded pomegranate varieties can be identified.

Here, we assembled a high‐quality draft genome sequence of the soft‐seeded ‘Tunisia’ pomegranate cultivar using single‐molecule real‐time (SMRT) sequencing and high‐throughput chromosome conformation capture (Hi‐C) techniques. We also resequenced 26 genetically diverse pomegranate varieties that varied regarding seed hardness and geographical distribution. Our comprehensive comparative genomic and population genetic analyses provided insights into the genetic diversity and population structure of pomegranates and are helpful to clarify the genetic divergence between soft‐ and hard‐seeded varieties. This genome sequence represents an unmatched resource for elucidating the genetic system and facilitating the molecular improvement of pomegranate.

## Results

### Genome sequencing and assembly

The Pacific Biosciences (PacBio) Sequel platform was used for the SMRT sequencing of the soft‐seeded ‘Tunisia’ genome. A total of 20.94 Gb PacBio long reads were used for assembly, resulting in a 320.31 Mb assembly (Table 1), which is close to the estimated size based on flow cytometry (Figure S1). The quality of the assembled genome was evaluated using three strategies. First, 168 396 276 Illumina paired‐end reads for the same pomegranate species were aligned to the genome assembly using the Burrows‐Wheeler Aligner (BWA) (Heng and Richard, 2010). A total of 96.76% of the reads were successfully aligned to the genome (Table S1). Second, according to BUSCO (version 2) (Simao *et al*., 2015), 93.33% (1344 of 1440) of the core eukaryotic genes, including 1292 single‐copy orthologs and 52 duplicated orthologs, were detected in our assembly (Table S2). Lastly, we used CEGMA (version 2.5) (Parra and Korf, 2007) to assess the completeness of our genome assembly. A set of 454 of 458 highly conserved core genes were detected in our assembly (Table S3). These results indicated that our pomegranate genome sequence was almost complete.

**Table 1:** ‘Tunisia’ draft genome statistics

Assembly feature	Statistic
Estimate of genome size by flow cytometry	313.18 Mb
Total PacBio reads	20.94 Gb
Assembly length	320.31 Mb
Chromosome number(2n)	2 × 8
Assembly % of chromosome	97.76
Number of contigs	661
Contig N50	4.49 Mb
Longest contig	14.77 Mb
Number of scaffolds	473
Scaffold N50	39.96 Mb
Longest scaffold	55.56 Mb
Repeat region % of assembly	50.93
Predicted gene models	33 594
Assembly genes of chromosome	32 538
Average gene length	2229 bp
Average exon length	263 bp
Average CDS length	1048 bp
GC content %	40.38

Our genome assembly comprised 661 contigs and 473 scaffolds, with the longest contig and scaffold being 14.77 and 55.56 Mb, respectively. The contig and scaffold N50 values were 4.49 and 39.96 Mb, respectively (Table 1). Regarding the assembly with Illumina reads, Qin *et al*. (2017) reported a contig N50 of 66.97 kb for ‘Dabenzi’, whereas Yuan *et al*. (2017) reported a contig N50 of 97 kb for ‘Taishanhong’. Notably, the length of contig N50 for the new ‘Tunisia’ reference genome was 67‐ and 46‐fold greater than that for the recently published ‘Dabenzi’ and ‘Taishanhong’ genomes, respectively.

To build pseudo‐chromosomes, Hi‐C libraries were constructed and 17.62 Gb of clean data corresponding to approximately 55× coverage of our pomegranate genome were obtained (Tables S4). Consequently, more than 95% of the reads were unambiguously located on the assembly. The unique mapped read pairs that aligned with the genome accounted for 63.55% of the total identified pairs (Table S4). Consequently, 97.76% (313.13 Mb) of the assembly was anchored on eight chromosomes (Table S5 and Figure S2), consistent with the number of pomegranate chromosomes detected by Masoud *et al*. (2005).

To further verify the Hi‐C assembly, we constructed a genetic map by sequencing 163 F_1_ individuals from a cross between ‘Tunisia’ and ‘Sanbai’ based on specific length amplified fragment (SLAF) sequencing. The final integrated map between the two parental genomes spanned 1200.01 cM with 1725 markers in eight linkage groups (LGs), consistent with the number of chromosomes in ‘Tunisia’ (Table S6 and Figure S2). Of the eight LGs, LG1 was the largest, with 271 markers covering a genetic distance of 197.32 cM, and LG5 was the smallest, with 101 markers spanning 123.81 cM. The average marker interval for the LGs ranged from 0.31 to 2.15 cM, with an overall average distance of 0.70 cM (Table S6). Regarding the gaps between markers, 95.59% were <5 cM. The max gap ranged from 11.70 cM (LG6) to 42.60 cM (LG3). All the SLAFs from the certain LG could be aligned to the single chromosome (Figure S3). Based on the contig orientation information of the draft genome of ‘Tunisia’, we compared the orders and directions of contigs anchored by the genetic map. The genetic map shared 103 anchored contigs with the anchored genome (Table S6). Of those contigs, 63 (61.17%) were in accordance with the placement orders, indicating a collinear relationship between these two anchorings (Figure S4). Our chromosome‐scale assembly was nearly 83 Mb larger than the existing ‘Dabenzi’ assembly (230.88 Mb chromosome‐scale reads), indicating that our genome sequence was substantially more complete.

### Genome annotation

**Figure 1:**
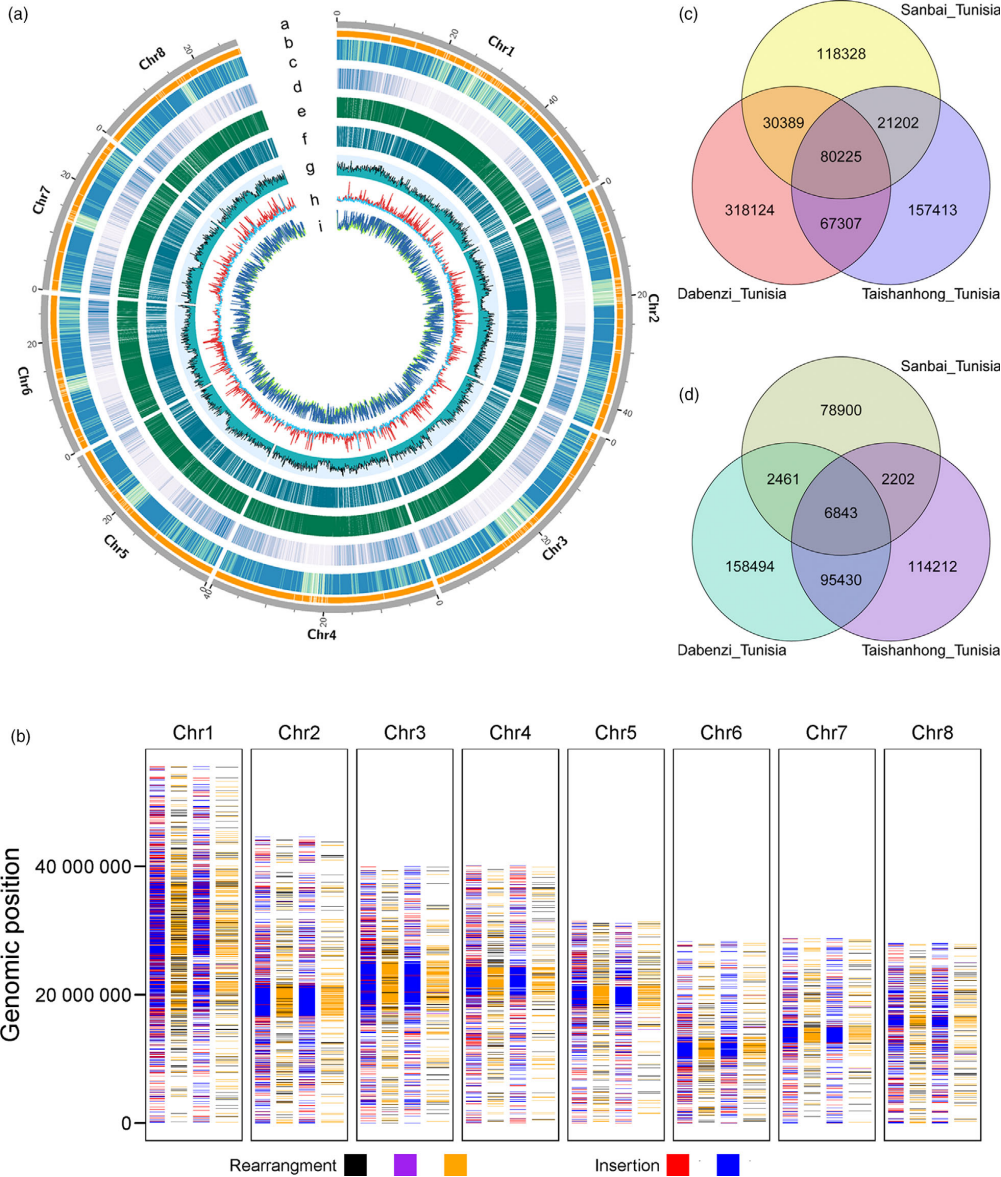
Characteristics of the ‘Tunisia’ genome and a global comparison of genomes. a, Rings represent: Chr, chromosome (i), gap (ii), repetitive elements (iii), gene density (iv), presence/absence variation (PAV) between ‘Tunisia’ and ‘Dabenzi’ (v), PAV between ‘Tunisia’ and ‘Taishanhong’ (vi), GC content (vii), insertion/deletion (InDel) and single nucleotide polymorphism (SNP) variations between ‘Tunisia’ and ‘Dabenzi’ (viii) and InDel and SNP variations between ‘Tunisia’ and ‘Taishanhong’ (ix). b, Rearrangements and inserted duplications in the Tunisia_Dabenzi (left) and Tunisia_Taishanhong (right) comparisons. Black, relocation; purple, inversion; orange, translocation; red, duplicated insertion; blue, other duplicated insertions. c, Venn diagram presenting the shared SNPs in the ‘Tunisia’, ‘Dabenzi’ and ‘Taishanhong’ genomes. d, Venn diagram presenting the shared InDels in the ‘Tunisia’, ‘Dabenzi’ and ‘Taishanhong’ genomes.

Predicted protein‐coding genes were annotated according to a combination of *ab initio*, homology‐based, and transcript evidence gathered from mixed‐tissue RNA sequences. A total of 33 594 high‐confidence protein‐coding gene models were predicted in the ‘Tunisia’ genome, with an average coding sequence length of 2229 bp and an average exon length of 263 bp (Table 1 and Figure 1a). Approximately 69.53% (23 357) of the genes were supported by transcriptome profiling using Illumina‐ and SMRT‐based RNA‐seq data with coverage > 0.7 and identity > 0.5 (Table S7). A total of 44 169 alternative splicing (AS) events were found to occur in eight chromosomes of ‘Tunisia’ based on the full‐length cDNA data (Figure S5).

A total of 28 080 (83.59%) protein‐coding genes were annotated based on the Gene Ontology (GO), Kyoto Encyclopedia of Genes and Genomes (KEGG), EuKaryotic Orthologous Groups (KOG), TrEMBL, nr and nt databases (Table S8). Additionally, 32 538 (96.85%) of the predicted ‘Tunisia’ genes were allocated to eight chromosomes (Table 1). Compared with previously published draft genomes, the ‘Tunisia’ genome has more genes. In addition to protein‐coding genes, 52 miRNA, 1468 rRNA, 440 tRNA and 1388 pseudogenes were identified in the ‘Tunisia’ pomegranate genome (Table S9).

Repetitive elements, which are major components of complex genomes, are widely dispersed throughout the genome and have multiple roles in genome evolution (Chalopin *et al*., 2015). Approximately 50.93% (163.12 Mb) of the ‘Tunisia’ assembly sequences were identified as repetitive elements (Figure 1a), including retrotransposons (Class I elements, 47.23%), DNA transposons (Class II elements, 4.57%), potential host genes (1.65%), simple sequence repeats (0.37%) and unclassified elements (8.42%) (Table S10). The proportion of Gypsy retrotransposon (17.33%) appears to have expanded considerably in the ‘Tunisia’ genome compared with that of the *Theobroma cacao* (9.00%). The proportion of full‐length long terminal repeats (LTR)/Copia repeats (7.20%) in the ‘Tunisia’ genome was similar to that in *Theobroma cacao* (7.00%). The PLE/LARD retrotransposon derivative repeat elements represented 17.86% of the genome, similar to the proportion of the genome represented by LTR/Gypsy elements (Table S10). In general, there was a higher proportion of repeats in the ‘Tunisia’ genome than in the ‘Dabenzi’ (155.3 Mb) and ‘Taishanhong’ (140.2 Mb) genomes.

### Global genome comparison analysis

Genomewide structural differences are important genetic factors underlying the observed phenotypic variations (Bianco *et al*., 2018; Ecker *et al*., 2009). When the chromosomes of ‘Tunisia’ were aligned to the ‘Taishanhong’ scaffolds, approximately 83.74% (268.26 Mb) of the ‘Tunisia’ genome sequence matched to 97.90% (268.30 Mb) of the ‘Taishanhong’ genome sequence in one‐to‐one syntenic blocks (Table S11). Similarly, we determined that 274.27 Mb (85.63%) of the ‘Tunisia’ genome sequence matched to 83.58% (274.49 Mb) of the ‘Dabenzi’ genome sequence in syntenic blocks (Table S11). Additionally, 59.72 and 58.64 Mb of the genome sequences were identified as duplicated insertions in the Tunisia_Dabenzi and Tunisia_Taishanhong comparisons, respectively. We detected 7242 and 1858 rearrangements (relocations, translocations and inversions), occupying 5.26 and 2.02 Mb of the genomes in the Tunisia_Dabenzi and Tunisia_Taishanhong comparisons, respectively (Tables S11 and Figure 1b). We randomly selected 108 structural variants (84 inserted duplications and 24 rearrangements) to carry out breakpoint analysis using clean subreads of PacBio (Table S12) and found that 80.56% of the breakpoints could be covered perfectly by PacBio reads. Nine and ten breakpoints were left and right unresolved, respectively. We subsequently identified 19 202 segments spanning 46.01 Mb of the ‘Tunisia’ genome that were absent in the ‘Taishanhong’ genome. We also identified 25 264 segments covering 41.13 Mb of the ‘Tunisia’ genome that were missing from the ‘Dabenzi’ genome (Table S11 and Figure 1a). As Illumina short sequencing reads are available for ‘Dabenzi’ (Qin *et al*., 2017), we aligned these reads to the 41.13 Mb genome regions and found that only 2765 segments (5.24 Mb) were supported by a clear reduction in read depth (Table S13 and Figure S6). These results further supported the more complete assembly of the ‘Tunisia’ genome constructed using the PacBio long reads.

Next, we examined the genomic genetic diversity between soft‐ and hard‐seeded cultivars. This genomewide comparison consisted of a systematic characterization of insertions/deletions (InDels) and single nucleotide polymorphisms (SNPs) among the ‘Taishanhong’, ‘Dabenzi’ and ‘Tunisia’ genomes (Figure 1c,d). In the Tunisia_Dabenzi comparison, we identified 496 045 SNPs and 263 228 small InDels, with an average of 1.5 and 0.8 per kb, respectively. In the Tunisia_Taishanhong comparison, we detected 326 147 SNPs and 218 687 small InDels, with an average of 1.0 and 0.7 per kb, respectively. Moreover, 147 532 SNPs and 102 273 InDels were common between the Tunisia_Dabenzi and Tunisia_Taishanhong comparisons. To further validate the InDels and SNPs, we completed the short‐read sequencing of the typical hard‐seeded cultivar ‘Sanbai’, which yielded 50 626 524 clean reads, with an average depth of 36× (Table S14 and Figure S7). These clean reads were then mapped to the ‘Tunisia’ genome. Accordingly, we identified 250 144 SNPs and 90 406 InDels, with an average of 0.8 and 0.3 per kb, respectively (Figure 1c,d). Of note, 80 225 SNPs and 6843 InDels were similar to those identified in the Tunisia_Dabenzi and Tunisia_Taishanhong comparisons (Figure 1c,d).

### Genes associated with SNPs and InDels

**Figure 2:**
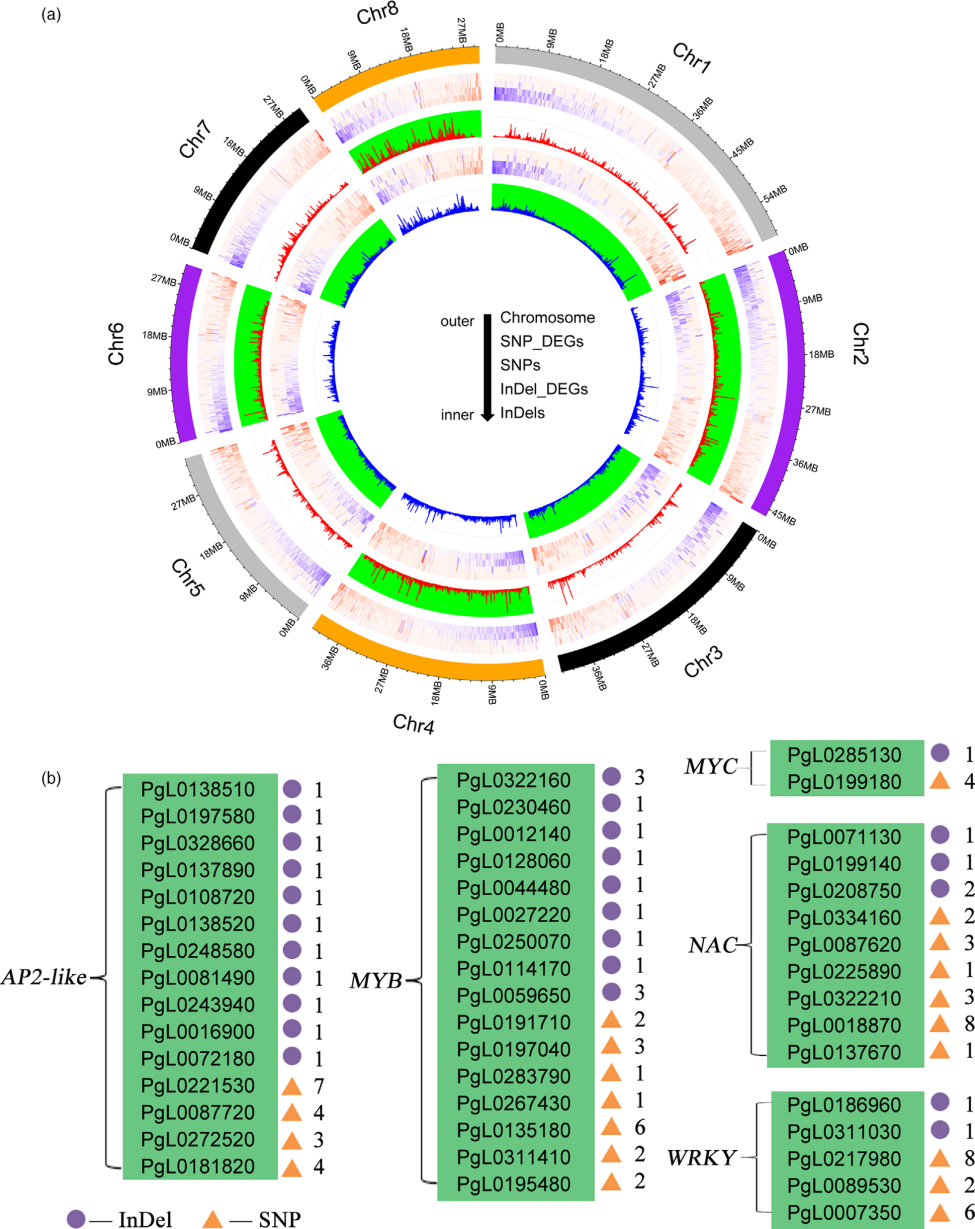
a, Distribution of the common SNPs and InDels detected in the Tunisia_Sanbai, Tunisia_Dabenzi and Tunisia_Taishanhong comparisons. Heatmap shows the differentially expressed genes based on the SNPs or InDels. b, Transcription factor families associated with different proportions of SNPs and InDels. Numbers represent the number of InDels or SNPs.

Genetic variations, including SNPs and InDels, are likely to lead to economically important traits (Saltz *et al*., 2017). A previous study demonstrated that an insertion in *PgLDOX* is responsible for the anthocyanin‐less phenotype of pomegranate fruit (Ben‐Simhon *et al*., 2015). According to the gene annotation results, the SNPs and InDels common among the Tunisia_Sanbai, Tunisia_Dabenzi and Tunisia_Taishanhong comparisons likely affect the functions of 13 034 genes in ‘Tunisia’. Transcriptomics of the seed revealed that of these genes, 3492 were highly differentially expressed between ‘Sanbai’ and ‘Tunisia’ during various seed developmental stages (Luo *et al*., 2018; Xue *et al*., 2017) (Table S15 and Figure 2a). These genes participate in several biological pathways, including cell division, cell wall biogenesis, signal transduction, transcriptional regulation, product transport and metabolism (Figure S8). The pathways related to cell division and the cell wall structure were proved to control pomegranate seed hardness (Luo *et al*., 2018; Niu *et al*., 2018). We also identified many transcription factor families, such as MYB (16 genes), WRKY (five genes), AP2‐like (16 genes), MYC (two genes) and NAC (nine genes), that contained different proportions of SNPs and InDels (Figure 2b). Most of these transcription factors have been reported to play roles in regulating the seed hardness of pomegranates (Luo *et al*., 2018; Xue *et al*., 2017) and hawthorns (Dai *et al*., 2013). In the comparison between ‘Tunisia’ and ‘Sanbai’, we detected a SNP (T‐C) at the 166‐bp position of the *NAC* (*PgL0137670*) transcription factor coding sequence. This SNP resulted in a nonsynonymous substitution, with lysine replaced by glutamic acid. A recent genetic analysis confirmed that the *PgL0137670* allelic variant (T) is associated with the soft‐seeded phenotype of pomegranate (Xia *et al*., 2019). Thus, the detected SNPs and InDels represent a valuable resource for studying pomegranate biology and molecular breeding.

### Genetic diversity and linkage disequilibrium decay

**Figure 3:**
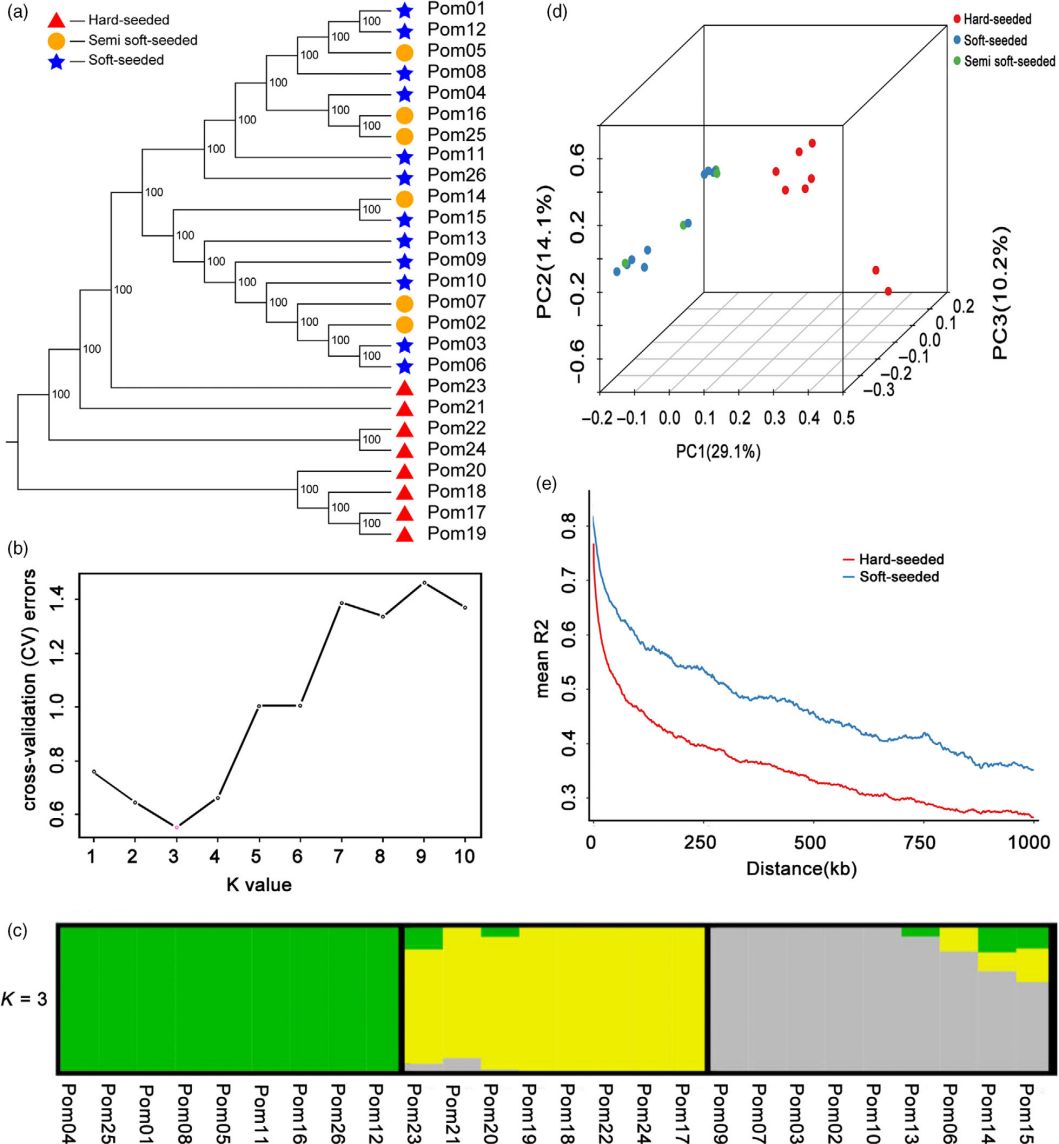
Genetic diversity and linkage disequilibrium decay among 26 pomegranate varieties. a, Phylogenetic relationships based on the genetic distance and population structure, presenting the distribution of K = 3 genetic clusters with the smallest cross‐validation error (b and c). d, Principal components for the variation among varieties. e, Linkage disequilibrium decay.

Genetic diversity and linkage disequilibrium decay (LD) were evaluated in natural populations to obtain a comprehensive overview of the polymorphisms correlated with phenotypic variations (Flint‐Garcia *et al*., 2003). In the current study, 26 pomegranate varieties, with various geographical origins and seed hardness, were collected. Resequencing the 26 genomes generated 191.24 Gb clean reads, with a median depth of 17 × and 97.91% coverage of the assembled genome. All clean reads were aligned to the ‘Tunisia’ genome, and a total of 4 882 740 SNPs were identified, with an average of 187 797 SNPs per genome (Table S16). The genomic positions of 77 876 of these SNPs were consistent with those of common SNPs among the three analysed cultivars (Table S17). We explored the phylogenetic relationships among the 26 pomegranate varieties based on 457 525 SNPs with a minor allele frequency > 0.05 and loci integrity ≥ 0.5. In the phylogenetic tree, the hard‐seeded varieties formed a subclade, and the soft‐ and semi soft‐seeded varieties were clustered within other subclades (Figure 3a). A population structure analysis indicated that the 26 varieties belonged to three subgroups (Q1, Q2 and Q3) (Table S16; Figure 3b,c). Eight hard‐seeded cultivars belonged to the Q2 subgroup, whereas the soft‐ and semi soft‐seeded cultivars were assigned to the Q1 and Q3 subgroups. A principal component analysis (PCA) revealed a similar population genetic structure, with hard‐seeded cultivars forming a tight cluster clearly separated from the others (Figure 3d). The top three PCs clearly separated these subpopulations and explained 28.09%, 12.92%, and 10.15% of the genetic variation in the pomegranate panel, respectively.

We analysed the LD to clarify the differences in the LD patterns in soft‐ and hard‐seeded populations. In general, the rate of LD decay varied among the chromosomes in both the hard‐ and soft‐seeded groups (Figure 3e). Additionally, the LD of the hard‐seeded group decayed significantly faster than the LD of the soft‐seeded group (*P *<* *0.01). This phenomenon may be due to consistent environmental conditions during cultivation (i.e. all of the soft‐seeded pomegranate varieties were adapted to the tropical Asian climate), which resulted in a narrow, or restricted, genetic background. Given the high LD in the soft‐seeded pomegranate varieties, only a small subset of SNPs would be required for marker‐assisted breeding. Collectively, the results of neighbour‐joining tree, population structure, PCA and LD analyses supported the clustering of the pomegranate clade based on seed hardness and may be useful for characterizing the divergence between soft‐ and hard‐seeded pomegranates.

### Selective sweep analysis

To compare the allelic diversity between soft‐ and hard‐seeded pomegranate groups, we completed a genomewide screening of nucleotide variations among the eight resequenced hard‐seeded genome sequences (H) from Q1 and the 12 soft‐seeded genome sequences (S) from Q2 or Q3 (Table S18). Calculating the divergence index (*F*_ST_) between soft‐ and hard‐seeded pomegranate varieties allowed us to identify genomic regions with high *F*_ST_ values, which indicated extensive diversification between the soft‐ and hard‐seeded pomegranate varieties. The average *F*_ST_ value was 0.26, and 131 nonoverlapping subregions were identified with *F*_ST_ values >0.55. These regions were distributed on all chromosomes and accounted for 4.4% of the ‘Tunisia’ genome. The average nucleotide diversity (π) of the H and S groups was 0.00047/kb and 0.00038/kb, with Tajima's *D* values of 1.12 and 1.00, respectively. These results revealed that the allelic diversity in the hard‐seeded population was generally higher than that in the soft‐seeded population across the entire genome.

The selective sweep regions usually contain, or are associated with, loci related to domestication, which may also contribute to the divergence between soft‐ and hard‐seeded pomegranate varieties. To detect the selective sweep signatures for domestication, we calculated the ratio of genetic diversity between the soft‐ and hard‐seeded pomegranate varieties across the whole genome and determined the cut‐off based on permutation tests (Figure 5). We then performed a whole‐genome screening of the overlapping selective sweep regions by combining the diversity ratios with the *F*_ST_. Finally, 14 and 10 selective sweep regions were identified in the soft‐ and hard‐seeded groups, respectively (Figure S9 and Table S19). According to the gene annotation result, the 14 and 10 selective sweep regions contained 282 and 249 loci in the soft‐ and hard‐seeded populations, respectively (Table S19).

### Selective genes mediating sucrose transport

**Figure 4:**
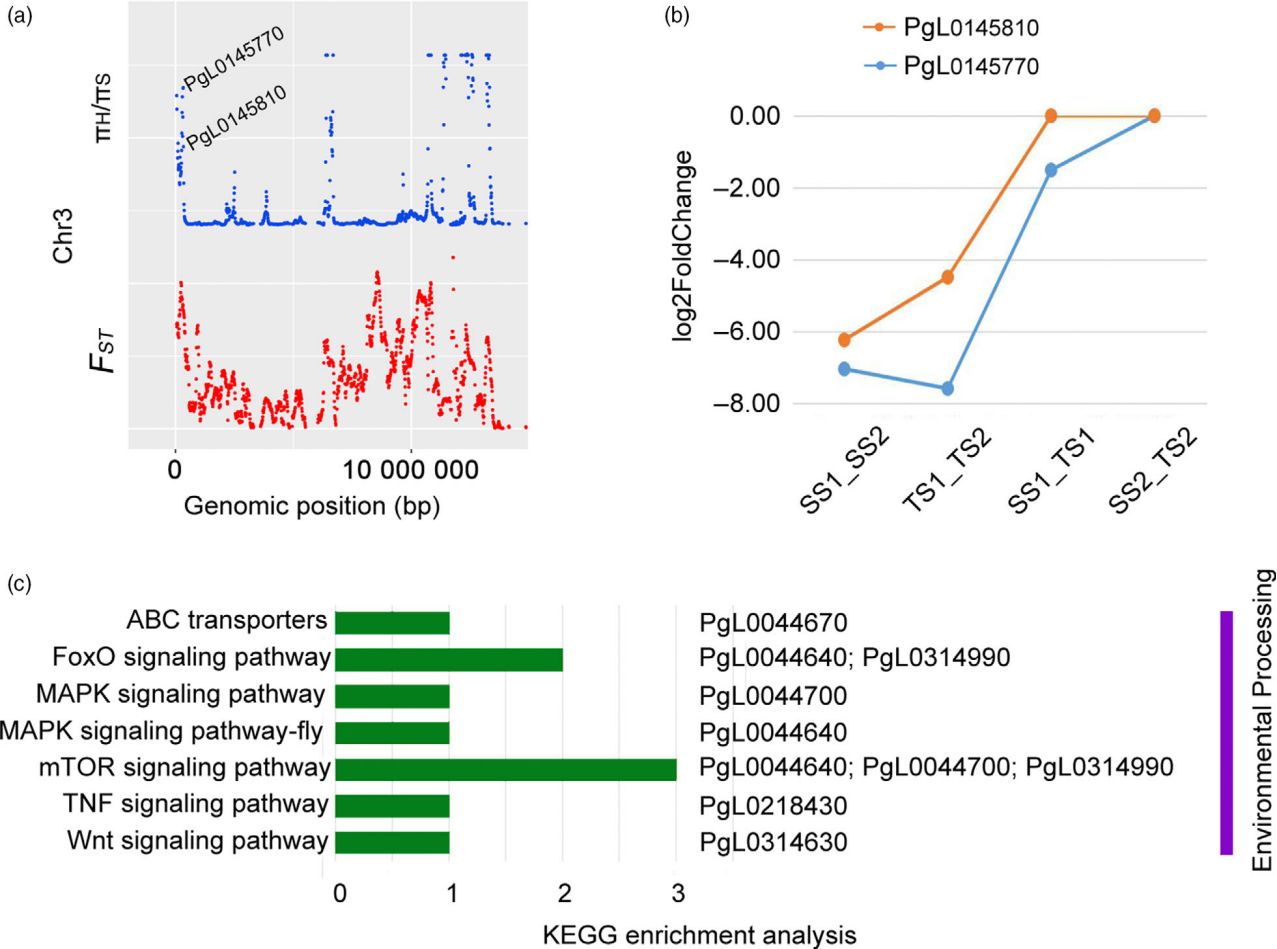
a, Mapping of the sucrose transport genes based on selective sweep analysis. π_H_/π_S_, nucleotide diversity ratio between hard‐ and soft‐seeded populations. b, Analysis of the differential expression of two sucrose transport genes at different pomegranate seed developmental stages. SS1_SS2, comparison between 60 and 120 days after flowering (DAF) for ‘Sanbai’ seeds; TS1_TS2, comparison between 60 and 120 DAF for ‘Tunisia’ seeds; SS1_TS1, comparison between ‘Sanbai’ and ‘Tunisia’ seeds at 60 DAF; SS2_TS2, comparison between ‘Sanbai’ and ‘Tunisia’ seeds at 120 DAF. c, Selective genes responsive to environmental conditions detected based on a KEGG enrichment analysis.

Sucrose allocation and transport in plants have been intensively studied for many years (Li *et al*., 2010, 2015). The seed yields of maize (Bezrutczyk *et al*., 2018) and rapeseed (Luo *et al*., 2015) depend on the efficient allocation of sucrose from the leaves to the seeds. Sucrose also functions as a signal in the regulation of strawberry fruit development and ripening (Jia *et al*., 2013). Sugar transport during seed development is also required for fibre elongation in cotton (Sun *et al*., 2018). On the basis of annotations in the ‘Tunisia’ genome, the *PgL0145770* and *PgL0145810* genes on chromosome 3 in the soft‐seeded population were determined to encode the sucrose transport proteins SUC8‐like and SUC6, respectively (Figure 4a). Seed transcriptome sequencing revealed that *SUC8‐like* and *SUC6* are more highly expressed at 60 days after flowering than at 120 days after flowering in both ‘Sanbai’ and ‘Tunisia’ (Luo *et al*., 2018), implying that these two genes are important for controlling seed development. Additionally, *SUC8‐like* significantly down‐regulated in ‘Tunisia’ compared to ‘Sanbai’ (*P *<* *0.05, Figure 4b), which may be related to differences in seed hardness between these two varieties. To the best of our knowledge, soft‐seeded pomegranate varieties normally have flourishing leaves, thick fruit peels and flesh, and only partially filled seeds. Down‐regulation of *SUC8‐like* expression in soft‐seeded pomegranate implies that photosynthetic products are not efficiently transported to seeds, ultimately resulting in soft seeds. This result greatly expands our understanding of the genetic mechanism underlying the development of soft pomegranate seeds.

### Selective genes mediating environmental adaptation

From an evolutionary perspective, environmental changes can result in selective pressure that favours the development of new traits (Scott, 2012). Mapping the selective genes that have become common in response to an environmental adaptation may help to characterize phenotypic divergence. A KEGG enrichment analysis indicated that in the hard‐seeded population, six selective genes (*PgL0314630*,* PgL0218430*,* PgL0044640*,* PgL0044700*,* PgL0044670* and *PgL0314990*) were enriched in the transporter or signalling pathways (Figure 4c). Specifically, *PgL0044640* and *PgL0314990* are involved in the forkhead box O (FoxO) signalling pathway*,* and *PgL0044700* is related to the mitogen‐activated protein kinase (MAPK) signalling pathway. Both FoxO and MAPK signalling pathways are involved in *Drosophila melanogaster* (Polesello and Bourg, 2017) and tomato fruit (Zhao *et al*., 2013) responses to cold conditions. These results may explain why hard‐seeded pomegranates are more tolerant to cold stress than soft‐seeded pomegranates.

### A large selective sweep region on chromosome 1

**Figure 5:**
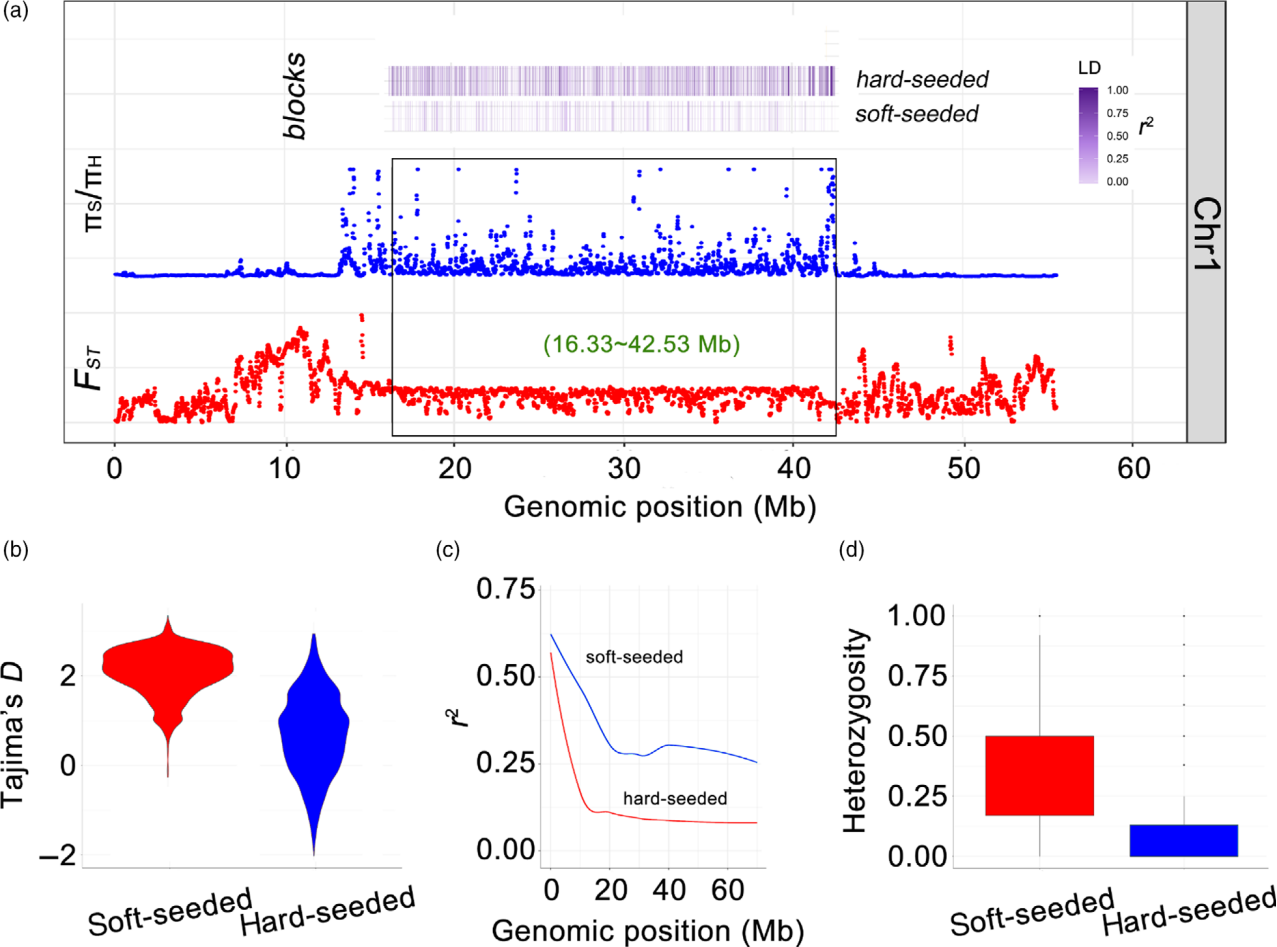
a, An exceptionally large selective sweep region detected on pomegranate chromosome 1. Black box, selective region; purple transitional rectangles, LD blocks in soft‐ and/or hard‐seeded populations; orange rectangles, haplotype blocks; π_S_/π_H_, nucleotide diversity ratio between soft‐ and hard‐seeded populations. b, Distribution of Tajima's *D* value in the selective region. c, Linkage disequilibrium decay determined based on the correlation (*r* ^2^) between allele frequencies and the distance between polymorphic sites in the selective region. Red line, soft‐seeded population; blue line, hard‐seeded population. d, Distribution of heterozygosity values in the selective region.

In the hard‐seeded population, we detected a 26.2 Mb (16.33–42.53 Mb) selective sweep region on chromosome 1 (Figure 5a). Within this region, we identified many loci with strong selection signals (π_S_/π_H_ > 8, mean Tajima's *D *=* *2.08) (Figure 5b). Additionally, the *F*_ST_ value for this region between the soft‐ and hard‐seeded populations was <0.30. Further analysis revealed that 23 397 SNPs with highly differentiated alleles were enriched in the 26.2 Mb region, accounting for 34.71% of all of the SNPs on chromosome 1. These SNPs were associated with a fast LD decay in the hard‐seeded group (Figure 5c). Furthermore, the 26.2 Mb region exhibited unusually low heterozygosity (average heterozygosity = 0.15) in the hard‐seeded group (Figure 5d). This extremely low recombination rate may have been due to large‐scale structural changes, including inversions, relocations and translocations, as described for the comparative genomic analysis (Figure 1b). Additionally, this 26.2 Mb region is near to the centromere, a structure that usually has a low recombination rate in diverse organisms, including humans (Choo, 1998) and pigs (Ai *et al*., 2015).

Our analyses suggested that the large selective sweep region may be the result of natural selection. First, the hard‐seeded populations were from China, whereas the soft‐seeded populations originated from the United States of America, Italy, Tunisia and other countries (Table S16). Geographical barriers limit hybridization. Additionally, to the best of our knowledge, pomegranate has an extremely short history of extensive breeding and artificial selection, and there is no sexual barrier between soft‐ and hard‐seeded pomegranate varieties.

## Discussion

The lack of a high‐quality draft genome has been the main limiting factor for accelerating forest tree improvement. In the present study, a high‐quality chromosome‐scale genome assembly was produced for the soft‐seeded pomegranate ‘Tunisia’ based on SMRT sequencing and Hi‐C technologies. The finished genome assembly represents a noticeable improvement over previously published draft genomes regarding completeness and accuracy (Qin *et al*., 2017; Yuan *et al*., 2017). Therefore, the ‘Tunisia’ reference genome sequence described herein should be an useful resource for evolutionary and functional studies as well as for the genetic improvement of pomegranate varieties.

The divergence between hard‐ and soft‐seeded pomegranates is affected by two key factors: genomic variations and selective genes. Genomewide comparative analyses revealed many differences between hard‐ and soft‐seeded pomegranate varieties. These variations have critical effects on the phenotypic divergence of pomegranate. Additionally, high‐coverage short‐read sequencing was used to conduct population genomic analyses of 26 pomegranate varieties. We identified a genomewide set of selective genes, which will be valuable for evolutionary and selective analyses, particularly for identifying functionally important variations related to the genetic divergence of soft‐ and hard‐seeded pomegranates. The considerable variation and abundance of selective genes will also be useful for future genomewide association studies and for accelerating the gene clone of pomegranate. Some of the selective genes determined seed hardness differ from those related to cold tolerance, implying that it is possible to breed new pomegranate cultivars that are both freezing tolerance and soft‐seeded by artificial hybridizations or genetic manipulations. A large selective sweep signal on chromosome 1 detected in this study provides another attractive candidate region for further investigation for the genetic factors contributing to the divergence between hard‐ and soft‐seeded pomegranate varieties. Collectively, these sequenced cultivars are valuable resources for population genomics, and studies on the genetic variations among them are helpful for accelerating genetic gains in pomegranate breeding in future.

## Experimental procedures

### Plant materials

Pomegranate varieties were cultivated in a nursery at the Zhengzhou Fruit Tree Research Institute, Chinese Academy of Agricultural Sciences, Henan province, China. An 11‐year‐old *P. granatum* ‘Tunisia’ tree was used for sequencing to assemble the draft genome with SMRT sequencing technology. Additionally, all cultivars underwent short‐read sequencing with the Illumina HiSeq 2500 platform. Young leaves were collected from each cultivar and immediately frozen in liquid nitrogen before extracting nucleic acids.

### Genome size estimation by flow cytometry

The DNA extracted from ‘Tunisia’ leaves was quantified by flow cytometry with a MoFlo XDP Cell Sorter (Beckman Coulter, Miami, FL). Specifically, the DNA quantity was calculated as the ratio of the pomegranate P1 peak fluorescence to maize (internal reference) P2 peak fluorescence.

### Library construction and sequencing

Genomic DNA was extracted using an established CTAB protocol (Murray and Thompson, 1980). Sequencing libraries were constructed according to the manufacturer's protocol (Illumina, San Diego, CA). Reads were trimmed to remove adaptors and filtered to eliminate low‐quality reads. More than 5 μg sheared and concentrated ‘Tunisia’ DNA was used to construct a SMRTbell library with the PacBio DNA Binding Kit 2.0 following the manufacturer's protocol. The library was sequenced with P2 DNA polymerase and the PacBio Sequel system. A total of four SMRT cells were sequenced for ‘Tunisia’, producing more than 20 Gb raw data.

### Genome assembly and contig anchoring

Raw PacBio reads were filtered by removing low‐quality reads, adaptor sequences and possible contaminated reads (i.e. of bacterial and viral origin). *De novo* assembly of the high‐quality PacBio reads was completed with the default parameters of the Canu pipeline (Koren *et al*., 2017). The resulting assembly was polished with Illumina PCR‐free 2 × 270 bp reads mapped with the BWA (version 0.7.10‐r789) (Heng and Richard, 2010) and the Pilon (version 1.22) program (parameters: –mindepth 10 –changes –threads 4 –fix bases) (Walker *et al*., 2014), which enabled the correction of 2754 SNPs, 741 insertions and 1531 deletions in the ‘Tunisia’ genome.

The Hi‐C libraries were prepared for ‘Tunisia’ roots, stems, leaves, flowers, fruit peels and seeds by the BioMarker Technologies Company (Beijing, China), as previously described (Xie *et al*., 2015). Briefly, nuclear DNA was cross‐linked and then cut with a restriction enzyme, leaving pairs of distally located but physically interacting DNA molecules attached to one another. The sticky ends of these digested fragments were biotinylated and then ligated to each other to form chimeric circles. Biotinylated circles (i.e. chimeras of the physically associated DNA molecules from the original cross‐linking) were enriched, sheared and sequenced. After filtering reads, we obtained 31.74 M valid interacting pairs for the chromosome‐level assembly of the ‘Tunisia’ genome. The contigs within the assemblies were separately broken into 50‐kb fragments and were then clustered with the LACHESIS program based on the valid interacting read pairs.

Total RNA was extracted from the ‘Tunisia’ roots, stems, leaves, flowers, fruit peels and seeds with TRIzol reagent (Invitrogen, Carlsbad, CA) according to the manufacturer's instructions. The RNA integrity was checked by electrophoresis on a 0.8% denaturing formaldehyde gel. High‐quality RNA (15 μg) was reverse‐transcribed into cDNA with the SMARTer^™^ PCR cDNA Synthesis Kit optimized for preparing full‐length cDNA. The BluePippin^™^ Size Selection System (Sage Science, Beverly, MA) was used for size fractionation and selection (1–2 kb, 2–3 kb, and > 3 kb). The SMRTbell libraries were constructed with the PacBio DNA Binding Kit 2.0, after which they were sequenced with P2 DNA polymerase and the PacBio Sequel system. Two SMRT cells were sequenced, and high‐quality sequences were acquired via a previously described three‐stage analysis of the full‐length transcriptome (Jiang *et al*., 2018).

### SLAF library preparation, sequencing, genotyping and genetic map construction

A total of 163 F_1_ individuals were derived from a cross between ‘Tunisia’ (soft‐seeded female parent) and ‘Sanbai’ (hard‐seeded male parent). Genomic DNA was extracted from the parents and progeny. We used SLAF sequencing to construct the genetic map. The library was constructed as previously described (Sun *et al*., 2013). Genomic DNA was digested with two enzymes (HaeIII and Hpy166II). After ligating the duplex tag‐labelled adaptor (PAGE‐purified; Life Technologies, Carlsbad, CA) and amplifying by PCR, the amplicons were purified and loaded onto agarose gels. Fragments ranging in size from 364 to 414 base pairs (with indexes and adaptors) were excised, purified and sequenced with the Illumina HiSeq 2500 platform (Illumina) according to the manufacturer's recommendations.

The SLAF marker identification and genotyping were performed as described by Sun *et al*. (2013). Briefly, low‐quality reads (quality score < 20e) were filtered out and the remaining raw reads were sorted for each progeny according to duplex barcode sequences. After the barcodes and the terminal 5‐bp positions were trimmed from the high‐quality reads, the clean reads were clustered based on sequence similarities exceeding 90%. Clustered sequences were defined as one SLAF locus (Zhang *et al*., 2015). The SNPs in each SLAF locus between parents were then detected, and SLAFs with more than three SNPs were eliminated. The alleles of each SLAF locus were then defined according to parental reads with a sequence depth > 10‐fold, whereas for each offspring, the reads with a sequence depth > 2‐fold were used to define alleles. Because pomegranate is a diploid species, SLAF loci with more than four alleles were defined as repetitive SLAFs and discarded. Only SLAFs with two to four alleles were identified as polymorphic and considered potential markers. All polymorphic SLAF loci were genotyped according to the consistency of the parental and offspring SNP loci. The marker code of the polymorphic SLAFs was analysed based on the F_1_ population type.

Markers exhibiting distorted segregation (*P *<* *0.05) as well as markers with > 30% missing genotype data or an average sequencing depth < 2‐fold in each progeny and < 10‐fold in the parents were filtered. The aa × bb segregation pattern markers were then selected to construct a genetic linkage map. Specific‐locus amplified fragments were partitioned primarily into different LGs based on a pairwise modified logarithm of odds score > 11. The HighMap strategy (Liu *et al*., 2014) was used to order the SLAF markers and correct genotyping errors within LGs. The SMOOTH algorithm was then adopted to correct any remaining genotyping errors according to the parental contributions to the genotypes (Os *et al*., 2005). Additionally, the k‐nearest neighbour algorithm was applied to impute missing genotypes (Huang *et al*., 2012). Skewed markers were then added to this map by applying a multipoint maximum‐likelihood method. Map distances were estimated with the Kosambi mapping function (Kosambi, 2016). The contigs anchored by the genetic map were located to the assembled ‘Tunisia’ genome by using ALLMAPS software (Tang *et al*., 2015).

### Prediction of repetitive elements

The pomegranate repeat sequences were identified using a combination of *de novo* and homology‐based searches with Repbase (Jurka *et al*., 2005). Several *de novo* prediction programs, including MITE‐Hunter (Han and Wessler, 2010), LTR‐FINDER (Zhao and Hao, 2007), RepeatScout (Price *et al*., 2005) and the PILER‐DF repetitive domain prediction program (Edgar and Myers, 2005), were used to construct a *de novo* repeat library. The PASTEClassifier (version 1.0) program (Claire *et al*., 2014) was used to classify the repeats and then combine with those in the Repbase (version 19.06) database (Jurka *et al*., 2005). Moreover, RepeatMasker (version 4.0.6) (Tamura, 1992) was used to identify and classify the repeat elements in the ‘Tunisia’ genome.

### Gene predictions and functional annotations

Protein‐coding genes were predicted with homology‐based, *de novo* and transcript‐based approaches. Open reading frames within the transcripts were predicted with the TransDecoder program (version 2.0). *Arabidopsis_thaliana, Sorghum_bicolor*,* Malus* × *domestica*,* Vitis_vinifera* and *Eucalyptus grandis* were used for the homology‐based predictions with GeneMoMa (Keilwagen *et al*., 2016). Potential candidate gene sets generated by PASA (version 2.0.2) (Haas *et al*., 2003) based on cDNA sequences as well as homology‐based gene models were used for analyses with *de novo* prediction software packages (i.e. AUGUSTUS, SNAP, Glimmer, Genescan and GeneID). These programs were used to generate *de novo* gene models from masked and unmasked genome sequences. The outputs from three approaches were integrated with EVM (version 1.1.1) (Chen, 2004) to generate consensus gene models, which were then filtered to remove transposons and low‐confidence predictions. Additional alternative transcript isoforms were obtained from the Iso‐Seq data. Gene functions were assigned based on the best BLASTN (*e*‐value = 1e−5) matches for the alignments to the sequences in the National Center for Biotechnology Information nonredundant protein database (Aron *et al*., 2011) and the best matches for the BLASTP (*e*‐value = 1e−5) (Lobo, 2012) search of the TrEMBL database (Bairoch and Apweiler, 1997). Gene models were analysed with InterProScan (Zdobnov and Apweiler, 2001) to compile a list of INTERPRO domains and GO terms (Dimmer *et al*., 2012) for each predicted gene. Additionally, the genes were aligned (*e*‐value = 1e−5) to the sequences in the KEGG (Kanehisa and Goto, 2000) and the KOG (Tatusov *et al*., 2003) databases for an additional functional prediction and classification.

The miRBase, Rfam and tRNAscan‐SE (version 1.3.1) databases were used to predict noncoding RNAs (microRNAs, rRNAs and tRNAs) (Griffiths‐Jones *et al*., 2005; Nawrocki and Eddy, 2013). GenBlastA (version 1.0.4) (She *et al*., 2009) was used to identify homologous genome sequences, with the integrated gene set used as the query. Candidate genes with premature stop codons and frameshift mutations were selected as the final predicted pseudogenes with GeneWise (version 2.4.1) (Birney *et al*., 2004).

### Comparative genome analysis

The MUMmer (version 3.23) program (Delcher *et al*., 2002) was used to compare genomes (parameters: –maxmatch –c 90 –l 40). Syntenic blocks were filtered using the delta –filter –1 parameter and the one‐to‐one alignment block option. The putative PAVs were identified by extracting unaligned regions among ‘Tunisia’, ‘Taishanhong’ and ‘Dabenzi’ from the ‘show‐diff’ command in MUMmer. To identify putatively unique presence regions, 90 × short insert size library data from ‘Dabenzi’ (Qin *et al*., 2017) were aligned to ‘Tunisia’ using BWA (Heng and Richard, 2010). Sequences with depth of read coverage < 10× were selected as true absents for further analysis. We used show‐snp to identify SNPs and InDels in the one‐to‐one alignment block (parameter: –Clr TH). The effects of the SNPs and InDels were determined with snpEff (Cingolani *et al*., 2012). We used a sliding window method (window size, 100 kb; step, 10 kb) to calculate the distribution of SNPs and InDels along each chromosome. The circos diagram of SNPs and InDels distribution was drawn by using the software shinyCircos (Yu *et al*., 2017). The show‐diff function in MUMmer (version 3.23) (Delcher *et al*., 2002) was used for selecting unaligned regions and classifying them into three regions (‘link‐inversion’, ‘link‐jump’, and ‘gap’). We filtered unaligned sequences in gap regions and aligned the potentially unique regions to the ‘Tunisia’ genome using BLASTN (*e*‐value < 1e−5) and filtered regions with coverage > 50% and identity > 90% to obtain the final unique regions in the ‘Tunisia’ genome.

### Identification of inversions and translocations

The MUMmer (version 3.23) program (Delcher *et al*., 2002) was used to extract the alignment blocks with inversions and to filter the blocks with low similarity in the two flanking regions. We then manually checked the remaining inversion blocks and joined the neighbouring blocks within 50 bp. Translocations (i.e. DNA segment is differentially located in genomes) were detected by identifying noncolinear single‐copy homologous blocks (length > 100 bp; identity > 90%) among the genomes.

### Population resequencing and population structure analysis

The Illumina HiSeq XTen sequencing platform was used to generate the paired‐end reads of the population samples. Properly paired and uniquely mapped reads were then used to call SNPs. Reads containing 10 Ns were filtered before the alignment. The BWA (Heng and Richard, 2010) was used to map filtered reads back to the ‘Tunisia’ genome, with the following parameters: seed length of 32 bp, maximum three mismatches allowed in each read, and minimum aligned length of 35 bp to identify a mapped read. Reads that could not be properly paired or that resulted in multiple alignment sites were discarded. Additionally, duplicates were removed with Picard (http://sourceforge.net/projects/picard/). The SNPs of the population were called by GATK (McKenna *et al*., 2010), with no more than two SNPs in any 5‐bp window along the reference genome. Filtered SNPs were used to calculate the heterozygosity of each variety with the following equation: H (%) = [(number of heterozygous SNPs)/(number of total SNPs)] × 100.

The MEGA5 program (Tamura *et al*., 2011) was used to construct an unrooted neighbour‐joining tree based on the Kimura two‐parameter model, with 1000 bootstrap replicates. The Structure 2.3.4 software package (Pritchard *et al*., 2000) was used to determine the population structure based on SNPs and excluding the missing data or heterozygous genotypes. Ten independent runs were performed with K‐values (i.e. putative number of genetic groups) from 1 to 10. The length of the burn‐in period and the number of Markov chain Monte Carlo replications were set at 100 000. The most likely K‐value was determined according to the cross‐validation errors based on successive K‐values. Varieties with a probability of membership > 0.7 were assigned to the corresponding clusters, and those with a probability of membership < 0.7 were assigned to a mixed group. A PCA was completed with the SmartPCA program of the EIGENSOFT 6.0 software package (Price *et al*., 2006). The PCA was based on the whole‐genome SNPs, and the first three eigenvectors were plotted in three dimensions. The LD was calculated using the PLINK2 (version 1.90) software (Purcell *et al*., 2007). The overall genome LD was determined as follows: the correlation coefficient (*r* ^2^) of alleles was calculated for individual chromosomes using SNPs from the corresponding chromosome (parameter: –ld‐window‐r2 0–ld‐window 999999–ld‐window‐kb 1000), after which pairwise *r* ^2^ values were averaged across the whole genome. The LD decay was visualized for both hard‐ and soft‐seeded populations with ggplot2 (Wickham, 2016).

### Genetic index analysis

PopGenome (Pfeifer and Wittelsbuerger, 2012) was used to estimate the *F*_ST_, nucleotide diversity and Tajima's *D* for the whole genome of both hard‐ and soft‐seeded populations. Sliding windows of 100 kb with 10‐kb steps were used to calculate the *F*_ST_, nucleotide diversity, and Tajima's *D* across the whole genome. The top 5% of marks, defined by a sliding window, were selected as candidate selective sweep regions. We further integrated the adjacent selective sweep regions and annotated the corresponding genes across the ‘Tunisia’ genome.

### KEGG enrichment analysis

The differentially expressed genes were subjected to a KEGG pathway enrichment analysis using the tools available on the KOBAS2.0 website (http://kobas.cbi.pku.edu.cn/). We identified significantly enriched metabolic pathways using the hypergeometric test (http://en.wikipedia.org/wiki/Hypergeometric_distribution) and a corrected *P* value < 0.05.

### Accession codes

The ‘Tunisia’ pomegranate whole‐genome sequence and raw sequence data for the transcriptomes have been deposited in GenBank under a BioProject with accession number PRJNA565884 and PRJNA565888, respectively.

## Authors’ contributions

X.L. and S.C. designed the experiment. X.L. conceived the study and led the research together with H.X.L., Z.K.W., W.Y., P.Z., D.C., H.Y.Y., K.D.L., D.G.Z., F.H.Z., L.N.C., Q.W., D.J. and X.C.X. coordinated the sampling, bioinformatics and experimental work. X.L. interpreted the results and wrote the manuscript. Z.K.W., W.Y., P.Z., D.C. and P.K. revised the manuscript. All authors have read and approved the current version of the manuscript.

## Conflict of interest

The authors declare no conflicts of interest.

## Supporting information


**Figure S1** Pomegranate genome size estimated by flow cytometry. P1 and P2 refer to the pomegranate and maize fluorescence peaks, respectively. The estimated ‘Tunisia’ genome size was calculated as follows: 8606/60 455 × 2200 Mb = 313.18 Mb.
**Figure S2** Chromosome‐scale scaffold of the *de novo* ‘Tunisia’ genome assembly based on chromatin interactions.
**Figure S3** Anchoring of genetic markers to the eight chromosomes. The *X*‐axis represents the physical distance (top) and genetic length (down). Red bar, linkage map; blue bar, physical map.
**Figure S4** Collinear patterns between anchored contigs with genetic map (LG) and the anchored genome (Chr).
**Figure S5** Distribution of the number of alternative splicing events along each chromosome.
**Figure S6** Example of using short−read alignment to verify a missing region mapped in ‘Dabenzi’. Blue triangle represents short read.
**Figure S7** Genome‐wide SNP distribution in ‘Sanbai’ pomegranate (a). ‘Sanbai’ pomegranate InDel length distribution in the genome (right) and gene CDS (left) (b).
**Figure S8** Distribution of differentially expressed genes associated with SNP and InDel variations among different biological pathways.
**Figure S9** Genome‐wide distribution of the divergence index (*F*_ST_) value, nucleotide diversity (π), Tajima's *D* value, and the selective genes in soft‐ and hard‐seeded populations. From top to bottom: purple line, distribution of Tajima's *D* value in the soft‐seeded population; orange line, distribution of Tajima's *D* value in the hard‐seeded population; green line, distribution of nucleotide diversity in the soft‐seeded population, blue line, distribution of nucleotide diversity in the hard‐seeded population; red line, distribution of the *F*_ST_ value for the soft‐ and hard‐seeded populations; black rectangles, selective genes in soft‐ (top) and hard‐seeded (bottom) populations.
**Table S1** ‘Tunisia’ genome sequencing data derived from short‐read sequencing.
**Table S2** Analysis of the ‘Tunisia’ genome with Benchmarking Universal Single‐Copy Orthologs.
**Table S3** Analysis of the ‘Tunisia’ genome with CEGMA v2.5.
**Table S4** Hi‐C library details.
**Table S5** Hi‐C genome assembly details.
**Table S6** Genetic linkage following cross between ‘Tunisia’ and ‘Sanbai’.
**Table S7** List of genes supported by transcriptome profiling.
**Table S8** Gene annotation details.
**Table S9** RNA details.
**Table S10** Repeat sequence details.
**Table S11** Global comparison of ‘Taishanhong’, ‘Dabenzi’, and ‘Tunisia’ genomes.
**Table S12** Breakpoint analysis of structural variants using clean subreads of PacBio.

**Table S13** PAV regions supported by the short inserted reads.
**Table S14** ‘Sanbai’ genome sequencing data derived from short‐read sequencing.
**Table S15** Differentially expressed genes associated with SNPs and InDels.
**Table S16** Analysis of SNP variations and population structures in 26 pomegranate varieties.
**Table S17** Analysis of the SNPs in the population with genomic positions that are consistent with those of common SNPs among the three analysed cultivars.
**Table S18** Analysis of genome‐wide nucleotide variations between soft‐ and hard‐seeded pomegranate groups.
**Table S19** Genes in the selective sweep regions of the soft‐ and hard‐seeded pomegranate groups.
