# Supplementary material for: The pomegranate (Punica granatum L.) draft genome dissects genetic divergence between soft‐ and hard‐seeded cultivars
Source: Plant Biotechnol J. 2019 Nov 6;18(4):955–68. doi: 10.1111/pbi.13260 (PMC7061868; doi:10.1111/pbi.13260)
Supplement: Supplementary file 1 — Figure S1 Pomegranate genome size estimated by flow cytometry. P1 and P2 refer to the pomegranate and maize fluorescence peaks, respectively. The estimated ‘Tunisia’ genome size was calculated as follows: 8606/60 455 × 2200 Mb = 313.18 Mb. Figure S2 Chromosome‐scale scaffold of the de novo ‘Tunisia’ genome assembly based on chromatin interactions. Figure S3 Anchoring of genetic markers to the eight chromosomes. The X‐axis represents the physical distance (top) and genetic length (down). Red bar, linkage map; blue bar, physical map. Figure S4 Collinear patterns between anchored contigs with genetic map (LG) and the anchored genome (Chr). Figure S5 Distribution of the number of alternative splicing events along each chromosome. Figure S6 Example of using short−read alignment to verify a missing region mapped in ‘Dabenzi’. Blue triangle represents short read. Figure S7 Genome‐wide SNP distribution in ‘Sanbai’ pomegranate (a). ‘Sanbai’ pomegranate InDel length distribution in the genome (right) and gene CDS (left) (b). Figure S8 Distribution of differentially expressed genes associated with SNP and InDel variations among different biological pathways. Figure S9 Genome‐wide distribution of the divergence index (F ST) value, nucleotide diversity (π), Tajima's D value, and the selective genes in soft‐ and hard‐seeded populations. From top to bottom: purple line, distribution of Tajima's D value in the soft‐seeded population; orange line, distribution of Tajima's D value in the hard‐seeded population; green line, distribution of nucleotide diversity in the soft‐seeded population, blue line, distribution of nucleotide diversity in the hard‐seeded population; red line, distribution of the F ST value for the soft‐ and hard‐seeded populations; black rectangles, selective genes in soft‐ (top) and hard‐seeded (bottom) populations. Table S1 ‘Tunisia’ genome sequencing data derived from short‐read sequencing. Table S2 Analysis of the ‘Tunisia’ genome with Benchmarking Universal Sin [file PBI-18-955-s002.doc]

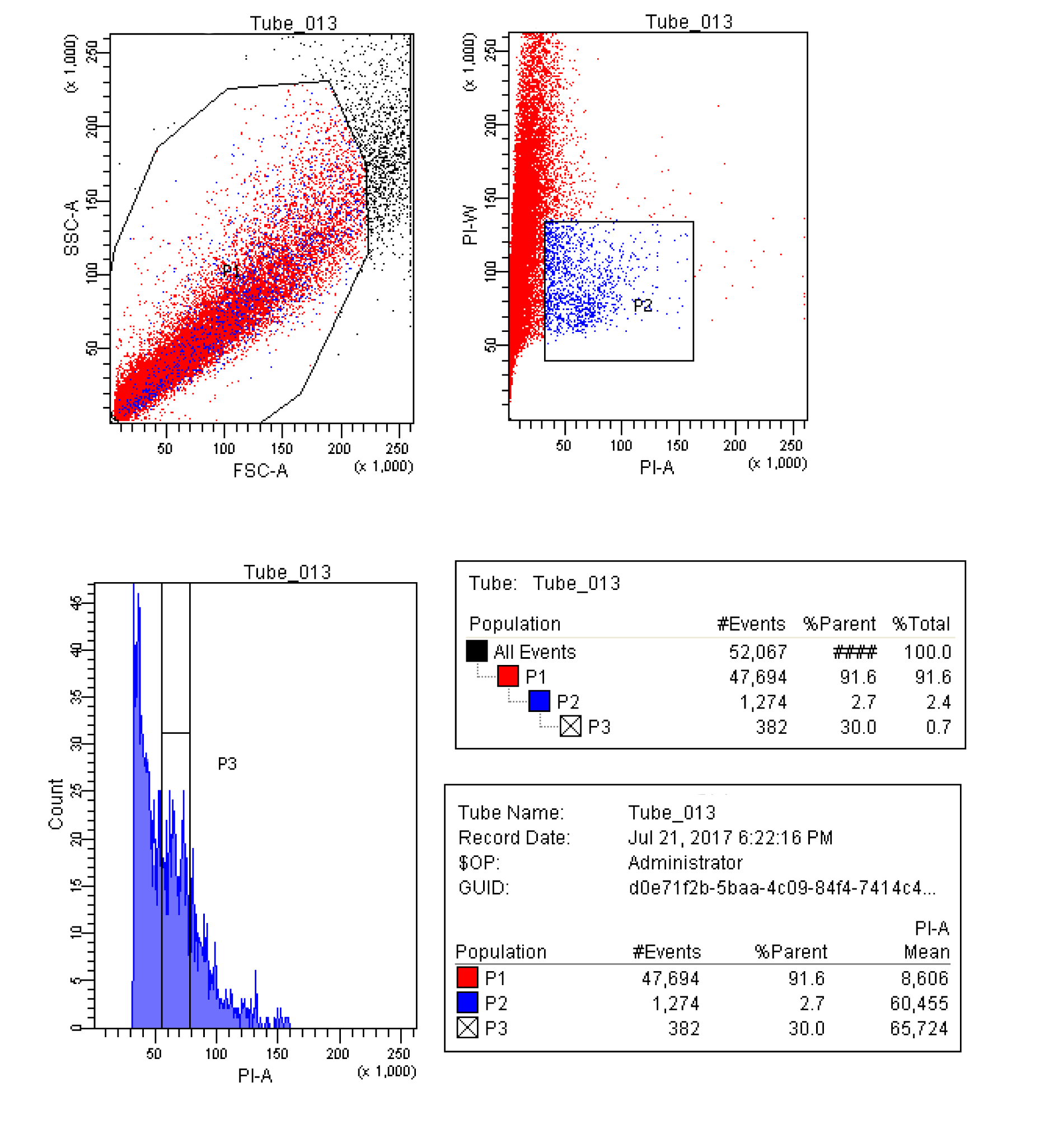


**Figure S1** Pomegranate genome size estimated by flow cytometry. P1 and P2 refer to the pomegranate and maize fluorescence peaks, respectively. The estimated ‘Tunisia’ genome size was calculated as follows: 8,606/60,455 × 2,200 Mb = 313.18 Mb.


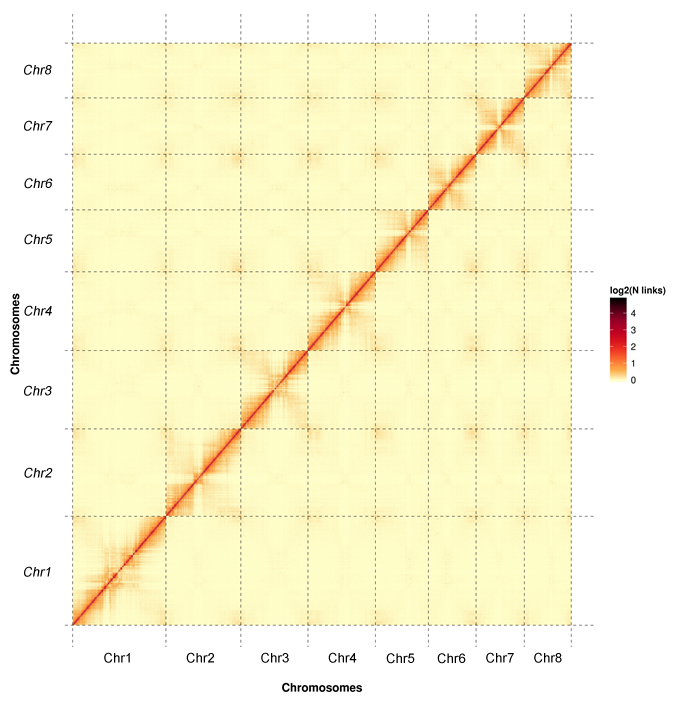


**Figure S2** Chromosome-scale scaffold of the *de novo* ‘Tunisia’ genome assembly based on chromatin interactions.


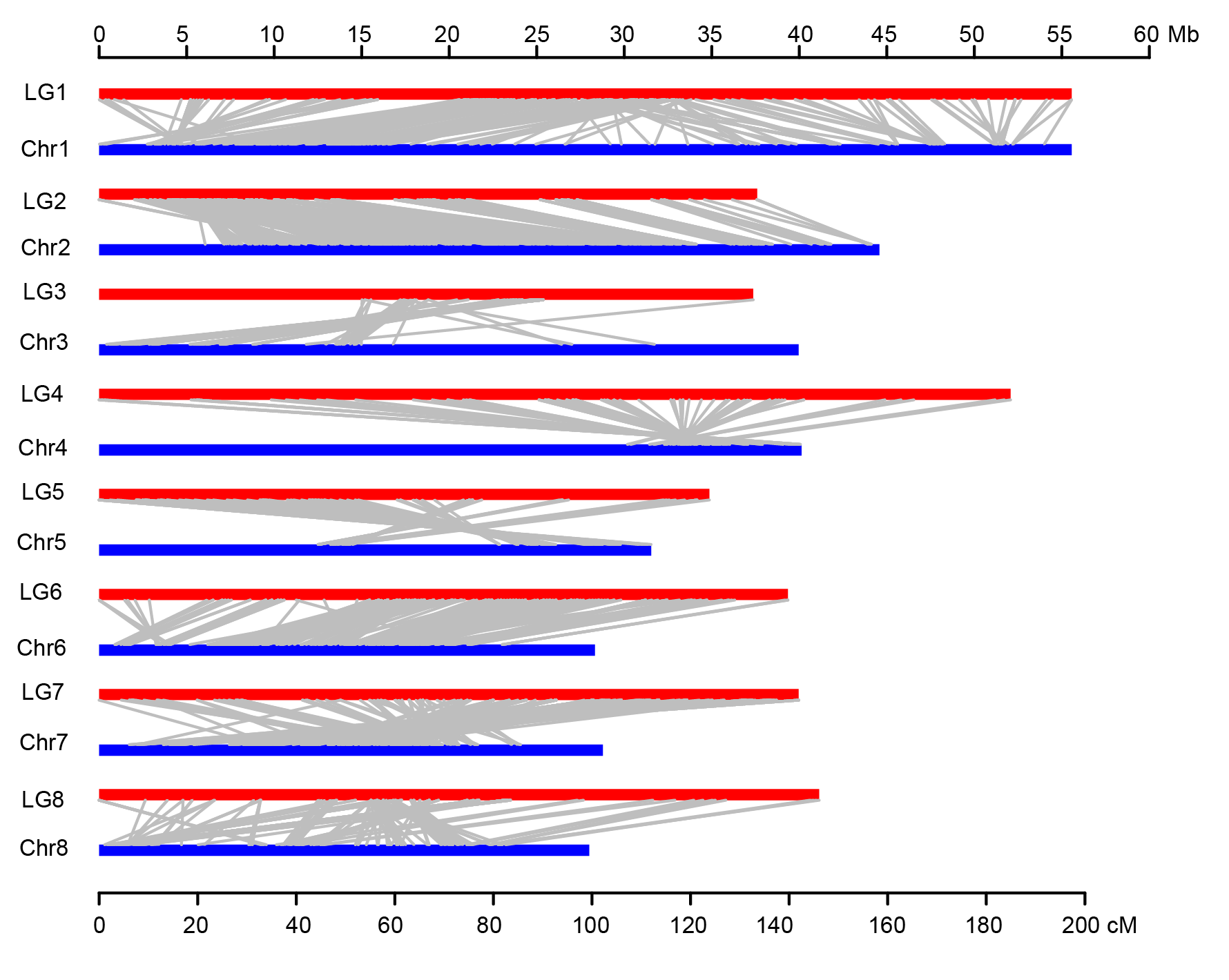


**Figure S3** Anchoring of genetic markers to the eight chromosomes. The X-axis represents the physical distance (top) and genetic length (down). Red bar, linkage map; blue bar, physical map.


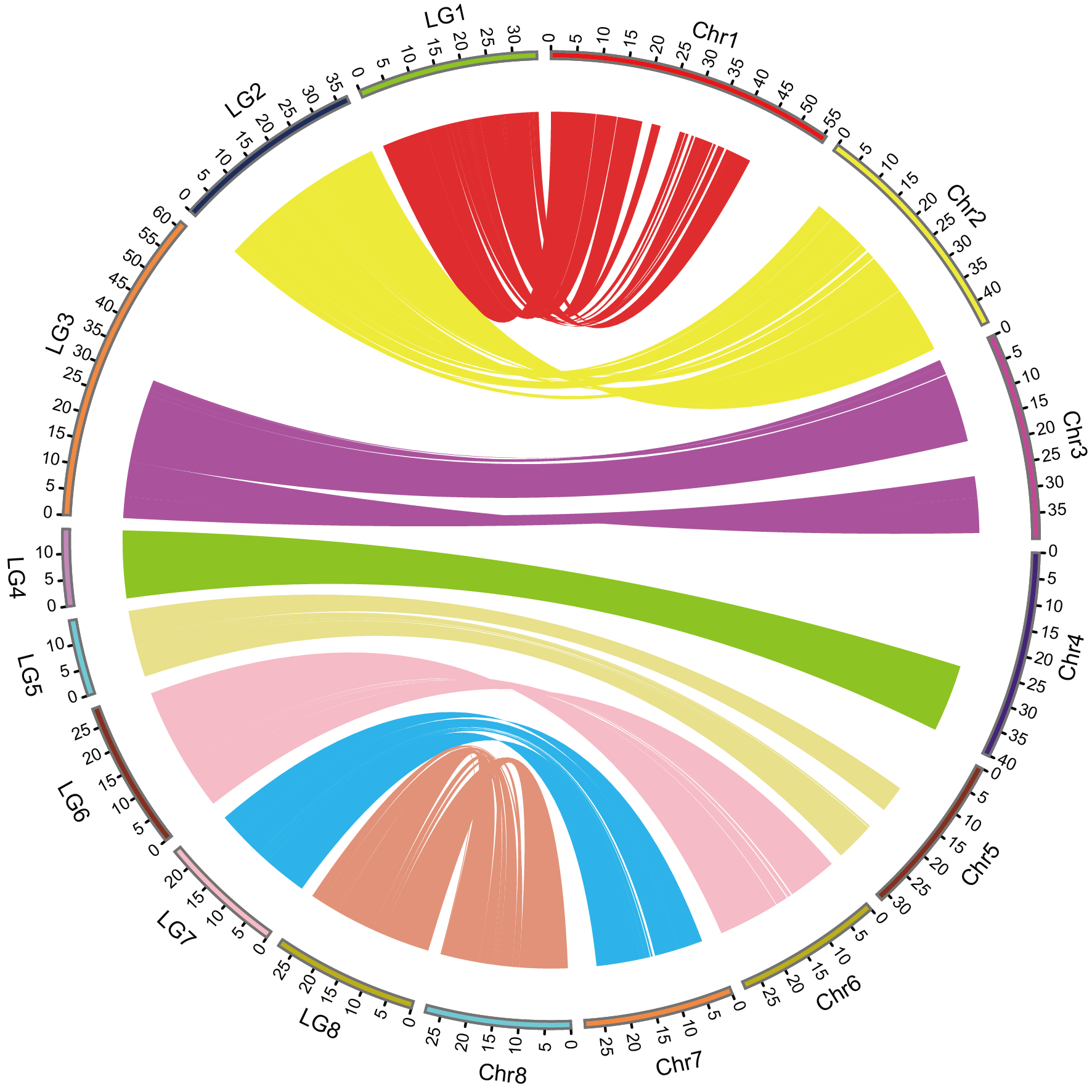


**Figure S4** Collinear patterns between anchored contigs with genetic map (LG) and the anchored genome (Chr).


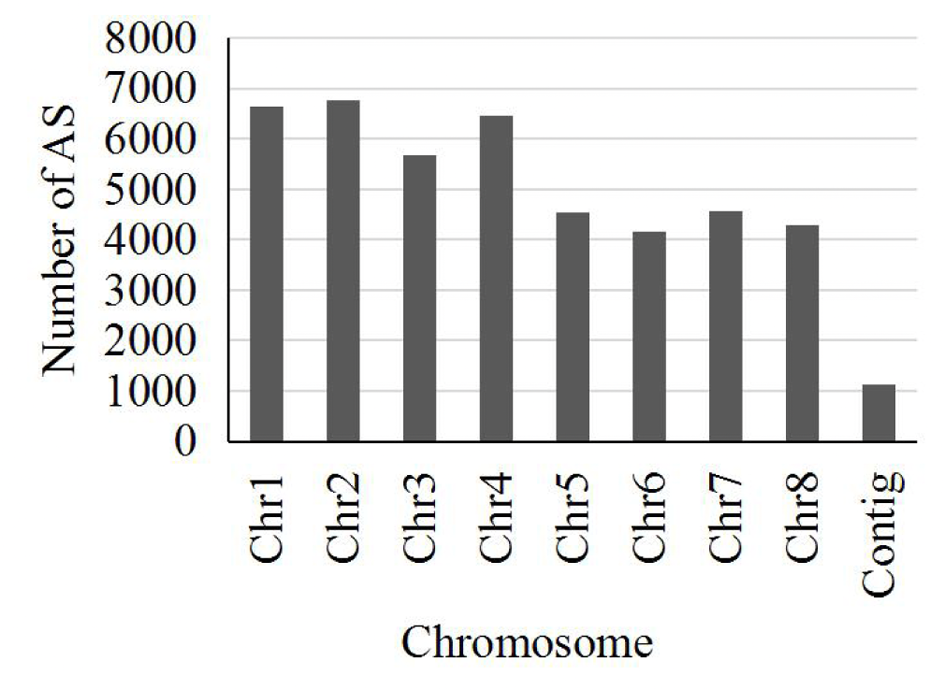


**Figure S5** Distribution of the number of alternative splicing events along each chromosome.


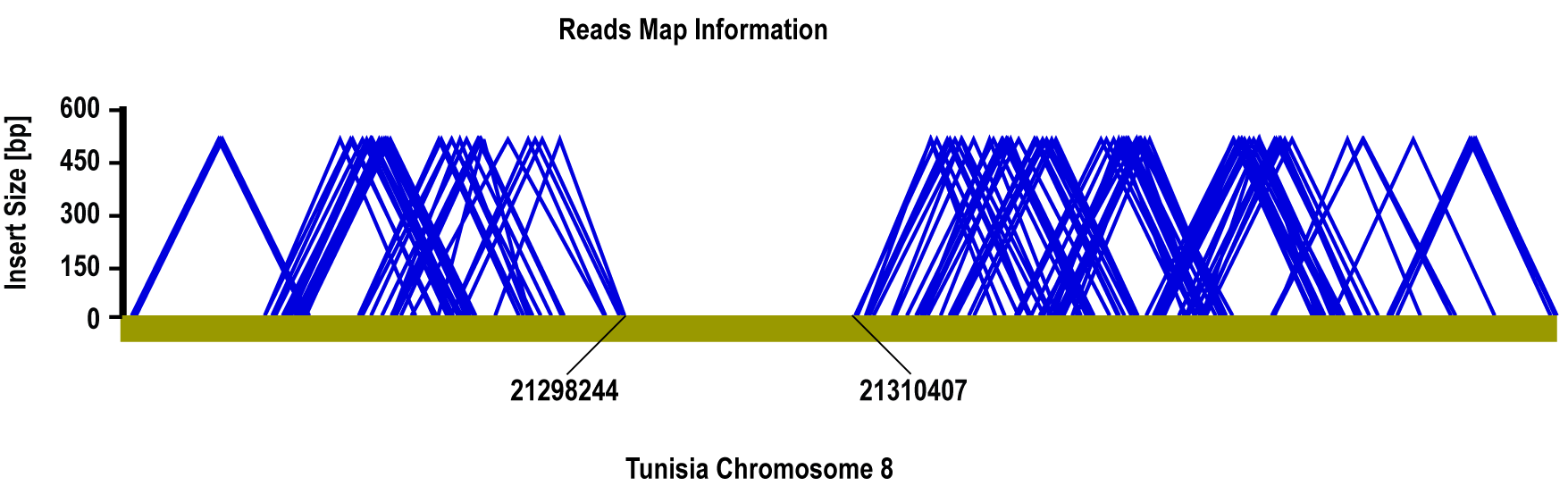


**Figure S6** Example of using short­read alignment to verify a missing region mapped in ‘Dabenzi’. Blue triangle represents short read.


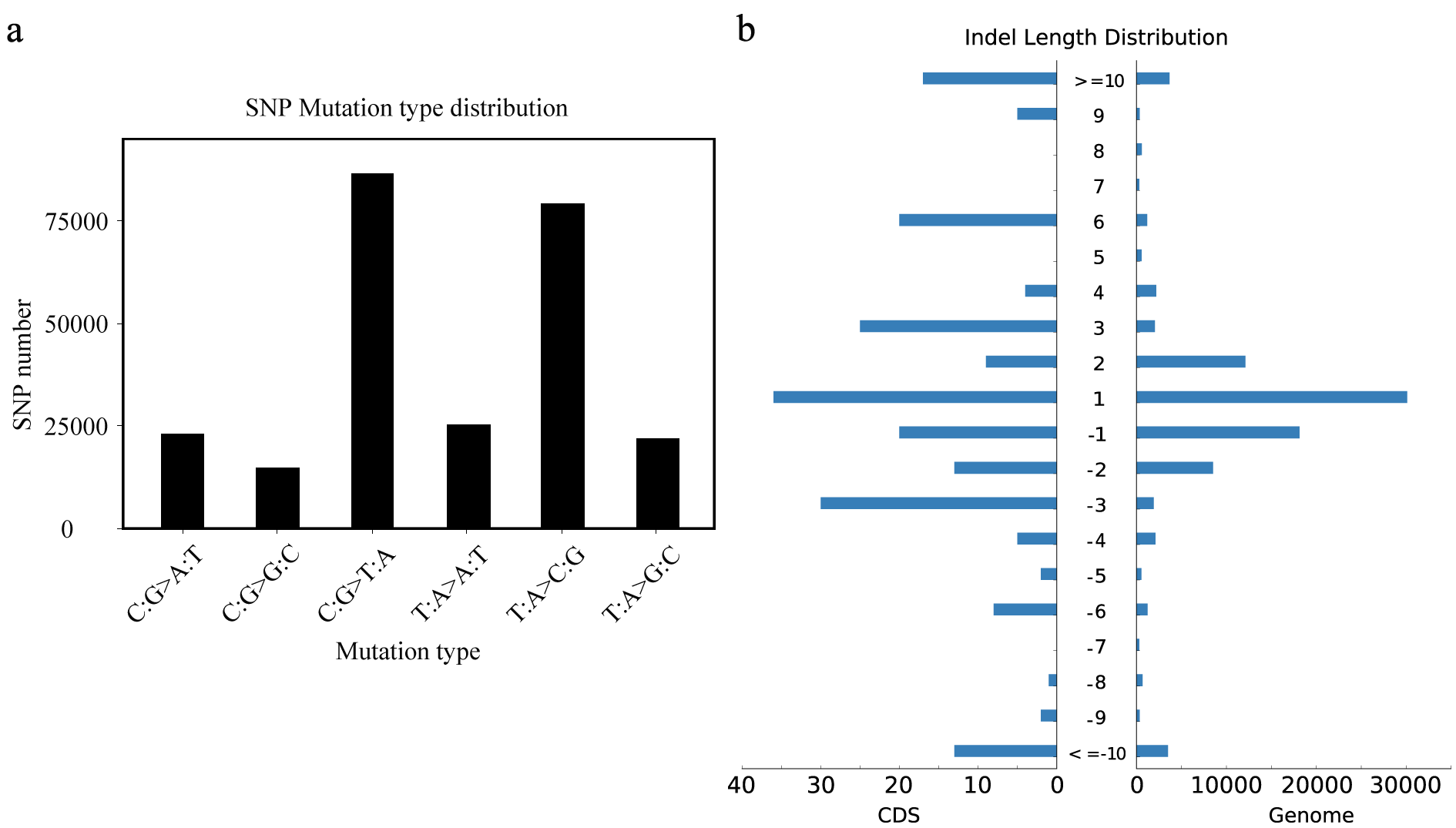


**Figure S7** Genome-wide SNP distribution in ‘Sanbai’ pomegranate (a). ‘Sanbai’ pomegranate InDel length distribution in the genome (right) and gene CDS (left) (b).


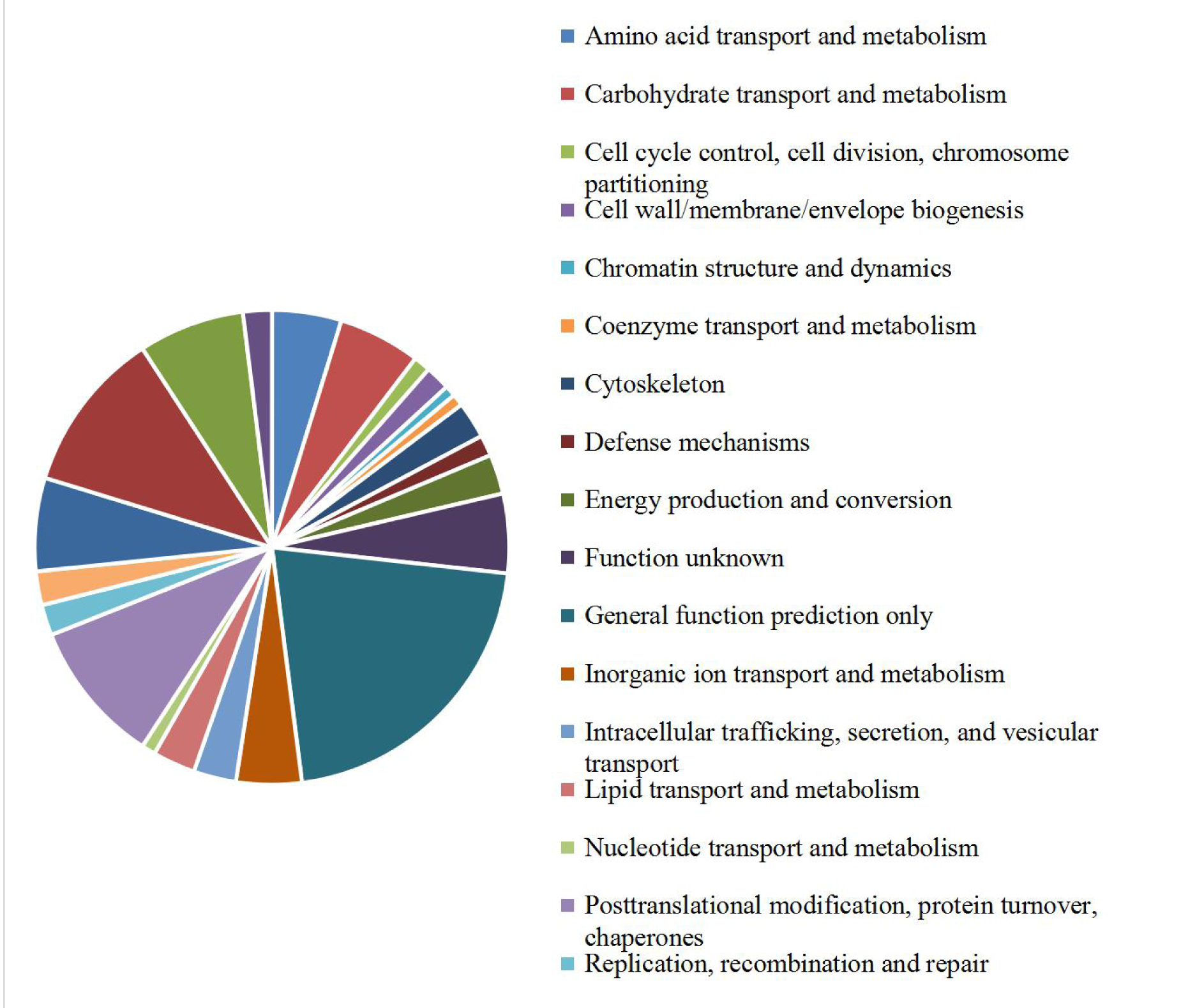


**Figure S8** Distribution of differentially expressed genes associated with SNP and InDel variations among different biological pathways.


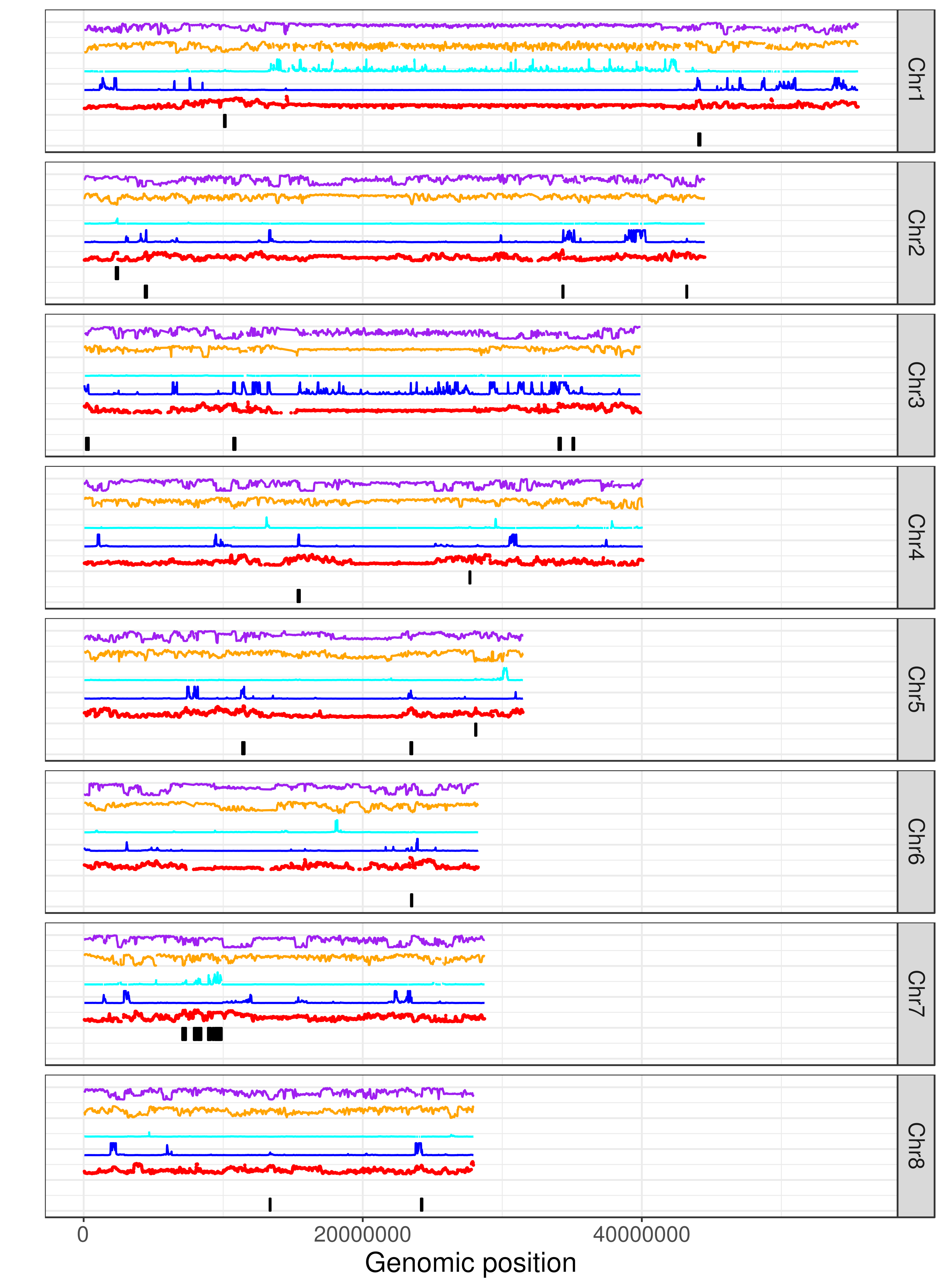


**Figure S9** Genome-wide distribution of the divergence index (*F_ST_*) value, nucleotide diversity (π), Tajima’s *D* value, and the selective genes in soft- and hard-seeded populations. From top to bottom: purple line, distribution of Tajima’s *D* value in the soft-seeded population; orange line, distribution of Tajima’s *D* value in the hard-seeded population; green line, distribution of nucleotide diversity in the soft-seeded population, blue line, distribution of nucleotide diversity in the hard-seeded population; red line, distribution of the *F_ST_* value for the soft- and hard-seeded populations; black rectangles, selective genes in soft- (top) and hard-seeded (bottom) populations.

**Supplementary Tables**

**Table S1** ‘Tunisia’ genome sequencing data derived from short-read sequencing

| **Sequencer** | **Library** | **Total reads** | **Mapped reads** | **Mapped (%)** | **Properly mapped reads** | **Properly_mapped (%)** |
| --- | --- | --- | --- | --- | --- | --- |
| Illumina | 270 bp | 168,396,276 | 167184518 | 99.28 | 162,941,710 | 96.76 |

**Table S2** Analysis of the ‘Tunisia’ genome with Benchmarking Universal Single-Copy Orthologs

| **Species** | **Complete BUSCOs** | **Complete and single-copy BUSCOs** | **Complete and duplicated BUSCOs** | **Fragmented** **BUSCOs** | **Missing BUSCOs** |
| --- | --- | --- | --- | --- | --- |
| Tunisia | 1,344 (93.33%) | 1,292 (89.72%) | 52 (3.61%) | 27 (1.88%) | 69 (4.79%) |

**Table S3** Analysis of the ‘Tunisia’ genome with CEGMA v2.5

| **Species** | **Number of 458 CEGs* present in assembly** | **% of 458 CEGs present in assemblies** | **Number of 248 highly conserved CEGs present** | **% of 248 highly conserved CEGs present** |
| --- | --- | --- | --- | --- |
| Tunisia | 454 | 99.12% | 248 | 100% |

**Table S4** Hi-C library details

| **Species** | **Base Number** | **Average depth** | **%≥Q30** | **Total Read Pairs** | **Mapped Reads (%)** | **Unique Mapped Read Pairs (%)** |
| --- | --- | --- | --- | --- | --- | --- |
| Tunisia | 17,622,990,702 | ~55× | 92.81 | 58,841,579 | 112,144,850 (95.29) | 37,391,666 (63.55) |

**Table S5** Hi-C genome assembly details

| **Group** | **Sequence Number** | **Sequence Length (bp)** | **Gene number** |
| --- | --- | --- | --- |
| Chr 1 | 115 | 60,204,099 | 5,069 |
| Chr 2 | 62 | 46,287,025 | 5,190 |
| Chr 3 | 50 | 41,709,127 | 4,184 |
| Chr 4 | 31 | 41,025,320 | 4,729 |
| Chr 5 | 50 | 33,541,134 | 3,574 |
| Chr 6 | 49 | 30,525,105 | 3,170 |
| Chr 7 | 44 | 30,196,088 | 3,402 |
| Chr 8 | 36 | 29,662,170 | 3,220 |
| Total genes |  |  | 32,538 |
| Total Sequences Clustered (Ratio %) | 437 (66.11) | 313,150,068 (97.76) |  |
| Total Sequences Ordered and Oriented (Ratio %) | 196 (44.85) | 296,829,111 (94.79) |  |

**Table S6** Genetic linkage following cross between ‘Tunisia’ and ‘Sanbai’

| **Linkage Group ID** | **Number of anchored contigs** | **Physical length (bp)** | **Total Marker** | **Total Distance (cM)** | **Average Distance (cM)** | **Gap < 5 cM** | **Max Gap (cM)** |
| --- | --- | --- | --- | --- | --- | --- | --- |
| LG1 | 1 | 14772832 | 271 | 197.32 | 0.73 | 97.05 | 16.58 |
| LG2 | 21 | 48838490 | 435 | 133.53 | 0.31 | 98.39 | 15.13 |
| LG3 | 26 | 38912757 | 100 | 132.71 | 1.33 | 95.00 | 42.60 |
| LG4 | 9 | 22748701 | 86 | 184.91 | 2.15 | 88.37 | 17.42 |
| LG5 | 17 | 27528477 | 101 | 123.81 | 1.23 | 95.05 | 19.16 |
| LG6 | 14 | 27934037 | 291 | 139.74 | 0.48 | 97.59 | 11.70 |
| LG7 | 8 | 30630512 | 269 | 141.93 | 0.53 | 98.51 | 12.28 |
| LG8 | 7 | 14633327 | 172 | 146.08 | 0.85 | 94.77 | 17.09 |
| Total | 103 | 225999133 | 1,725 | 1,200.01 | 0.70 |  |  |

**Table S8** Gene annotation details

| **Database** | **Annotated number** | **Percentage (%)** |
| --- | --- | --- |
| GO_Annotation | 14,532 | 43.26% |
| KEGG_Annotation | 8,426 | 25.08% |
| KOG_Annotation | 15,835 | 47.14% |
| TrEMBL_Annotation | 27,947 | 83.19% |
| nr_Annotation | 27,996 | 83.34% |
| All_Annotated | 28,080 | 83.59% |

**Table S9** RNA details

| **RNA classification** | **Number** | **Family** |
| --- | --- | --- |
| miRNA | 52 | 17 |
| rRNA | 1,468 | 4 |
| tRNA | 440 | 22 |
| Pseudogene | 1,388 |  |

**Table S10** Repeat sequence details

| **Type** | **Number** | **Length** | **Rate (%)** |
| --- | --- | --- | --- |
| ClassI/DIRS | 7125 | 8265334 | 2.58 |
| ClassI/LINE | 13180 | 4236065 | 1.32 |
| ClassI/LTR | 560 | 198144 | 0.06 |
| ClassI/LTR/Copia | 42659 | 23058754 | 7.20 |
| ClassI/LTR/Gypsy | 53128 | 55521079 | 17.33 |
| ClassI/PLE|LARD | 127648 | 57218832 | 17.86 |
| ClassI/SINE | 5930 | 1042642 | 0.33 |
| ClassI/SINE|TRIM | 4 | 1386 | 0 |
| ClassI/TRIM | 1484 | 1686401 | 0.53 |
| ClassI/Unknown | 333 | 71662 | 0.02 |
| ClassII/Crypton | 191 | 59925 | 0.02 |
| ClassII/Helitron | 16406 | 5716999 | 1.78 |
| ClassII/MITE | 2310 | 477093 | 0.15 |
| ClassII/Maverick | 1146 | 350005 | 0.11 |
| ClassII/TIR | 15725 | 7735272 | 2.41 |
| ClassII/Unknown | 3246 | 332072 | 0.1 |
| PotentialHostGene | 22317 | 5300588 | 1.65 |
| SSR | 1683 | 1183998 | 0.37 |
| Unknown | 61836 | 26979078 | 8.42 |
| Total with overlap: | 376911 | 199435329 | 62.26 |
| Total without overlap: | 376911 | 163120357 | 50.92 |

**Table S11** Global comparison of ‘Taishanhong’, ‘Dabenzi’, and ‘Tunisia’ genomes

| **Type** | **Tunisia_Dabenzi** | **Tunisia_Taishanhong** |
| --- | --- | --- |
| one-to-one length | 274.27 Mb | 268.26 Mb |
| Break points | 107,961 | 46,225 |
| Inserted duplications | 37,054 (59.72 Mb) | 23,379 (58.64 Mb) |
| Relocations | 1,664 (0.75 Mb) | 546 (0.47 Mb) |
| Translocations | 5,540 (4.5 Mb) | 1,284 (2.02 Mb) |
| Inversions | 38 (0.01 Mb) | 28 (0.03 Mb) |
| Total PAV | 25,264 | 19,202 |
| Total SNPs | 496,045 | 326,147 |
| Total Indels | 263,228 | 218,687 |

**Table S14** ‘Sanbai’ genome sequencing data derived from short-read sequencing

| **ID** | **Raw_Reads** | **Clean_Reads** | **Q20 (%)** | **Q30 (%)** | **Mapped ratio (%)** | **Ave_depth** |
| --- | --- | --- | --- | --- | --- | --- |
| Sanbai | 50,860,704 | 50,626,524 | 96.47 | 96.47 | 99.25 | 36 |

**Table S19** Genes in the selective sweep regions of the soft- and hard-seeded pomegranate groups

| **Chr** | **Gene_id** | **Start** | **End** | **Group** |
| --- | --- | --- | --- | --- |
| Chr1 | PgL0129990 | 44031550 | 44031864 | Soft_seeded |
|  | PgL0130000 | 44037512 | 44040478 | Soft_seeded |
|  | PgL0130010 | 44041887 | 44043878 | Soft_seeded |
|  | PgL0130020 | 44049951 | 44051681 | Soft_seeded |
|  | PgL0130030 | 44077368 | 44080088 | Soft_seeded |
|  | PgL0130040 | 44081746 | 44091717 | Soft_seeded |
|  | PgL0130050 | 44101686 | 44102246 | Soft_seeded |
|  | PgL0130060 | 44112570 | 44115137 | Soft_seeded |
|  | PgL0130070 | 44123101 | 44123868 | Soft_seeded |
|  | PgL0130090 | 44129268 | 44131576 | Soft_seeded |
|  | PgL0130100 | 44142060 | 44143703 | Soft_seeded |
|  | PgL0130110 | 44145042 | 44149890 | Soft_seeded |
|  | PgL0130120 | 44156304 | 44157669 | Soft_seeded |
|  | PgL0130130 | 44158711 | 44165645 | Soft_seeded |
|  | PgL0130140 | 44172979 | 44175681 | Soft_seeded |
|  | PgL0130150 | 44179838 | 44184426 | Soft_seeded |
|  | PgL0130160 | 44186718 | 44191317 | Soft_seeded |
|  | PgL0130170 | 44196531 | 44196950 | Soft_seeded |
|  | PgL0130180 | 44197999 | 44201646 | Soft_seeded |
|  | PgL0130190 | 44205364 | 44209907 | Soft_seeded |
| Chr2 | PgL0076420 | 34299977 | 34301412 | Soft_seeded |
|  | PgL0076440 | 34301544 | 34302594 | Soft_seeded |
|  | PgL0076460 | 34305999 | 34308239 | Soft_seeded |
|  | PgL0076470 | 34322860 | 34327556 | Soft_seeded |
|  | PgL0076480 | 34327579 | 34329182 | Soft_seeded |
|  | PgL0076490 | 34333215 | 34335526 | Soft_seeded |
|  | PgL0076500 | 34340934 | 34343767 | Soft_seeded |
|  | PgL0076510 | 34345020 | 34345872 | Soft_seeded |
|  | PgL0076520 | 34346509 | 34348328 | Soft_seeded |
|  | PgL0076530 | 34351313 | 34356441 | Soft_seeded |
|  | PgL0076540 | 34360933 | 34362823 | Soft_seeded |
|  | PgL0076550 | 34363423 | 34366831 | Soft_seeded |
|  | PgL0076560 | 34367774 | 34370661 | Soft_seeded |
|  | PgL0076570 | 34378423 | 34379160 | Soft_seeded |
|  | PgL0076580 | 34393201 | 34396299 | Soft_seeded |
| Chr2 | PgL0091030 | 43179488 | 43180516 | Soft_seeded |
|  | PgL0091040 | 43180634 | 43180954 | Soft_seeded |
|  | PgL0091050 | 43197442 | 43202975 | Soft_seeded |
|  | PgL0091060 | 43207451 | 43208400 | Soft_seeded |
|  | PgL0091070 | 43195166 | 43196904 | Soft_seeded |
|  | PgL0091080 | 43203254 | 43203640 | Soft_seeded |
|  | PgL0091090 | 43212854 | 43215321 | Soft_seeded |
|  | PgL0091100 | 43220263 | 43221948 | Soft_seeded |
|  | PgL0091110 | 43223977 | 43225906 | Soft_seeded |
|  | PgL0091120 | 43225966 | 43226358 | Soft_seeded |
|  | PgL0091130 | 43227418 | 43230674 | Soft_seeded |
|  | PgL0091140 | 43240679 | 43242178 | Soft_seeded |
|  | PgL0091150 | 43242590 | 43244854 | Soft_seeded |
|  | PgL0091160 | 43244986 | 43245882 | Soft_seeded |
|  | PgL0091170 | 43246223 | 43247624 | Soft_seeded |
|  | PgL0091180 | 43248121 | 43249936 | Soft_seeded |
|  | PgL0091190 | 43254281 | 43254958 | Soft_seeded |
|  | PgL0091200 | 43261667 | 43262302 | Soft_seeded |
| Chr2 | PgL0048070 | 4370203 | 4371925 | Soft_seeded |
|  | PgL0048080 | 4372041 | 4373609 | Soft_seeded |
|  | PgL0048090 | 4376278 | 4380440 | Soft_seeded |
|  | PgL0048100 | 4381985 | 4382708 | Soft_seeded |
|  | PgL0048110 | 4383076 | 4392173 | Soft_seeded |
|  | PgL0048120 | 4399740 | 4404119 | Soft_seeded |
|  | PgL0048130 | 4405566 | 4410337 | Soft_seeded |
|  | PgL0048140 | 4417253 | 4418151 | Soft_seeded |
|  | PgL0048150 | 4440352 | 4443346 | Soft_seeded |
|  | PgL0048160 | 4443745 | 4446339 | Soft_seeded |
|  | PgL0048170 | 4447224 | 4447908 | Soft_seeded |
|  | PgL0048180 | 4453785 | 4454466 | Soft_seeded |
|  | PgL0048190 | 4456332 | 4457348 | Soft_seeded |
|  | PgL0048200 | 4460975 | 4463915 | Soft_seeded |
|  | PgL0048210 | 4468192 | 4468923 | Soft_seeded |
|  | PgL0048220 | 4469648 | 4471214 | Soft_seeded |
|  | PgL0048230 | 4471901 | 4472314 | Soft_seeded |
|  | PgL0048240 | 4477665 | 4494913 | Soft_seeded |
|  | PgL0048250 | 4499495 | 4510852 | Soft_seeded |
|  | PgL0048260 | 4519273 | 4521135 | Soft_seeded |
|  | PgL0048270 | 4533439 | 4534453 | Soft_seeded |
|  | PgL0048280 | 4538673 | 4542130 | Soft_seeded |
|  | PgL0048290 | 4543214 | 4545373 | Soft_seeded |
|  | PgL0048300 | 4548196 | 4550089 | Soft_seeded |
| Chr3 | PgL0160050 | 10709052 | 10710575 | Soft_seeded |
|  | PgL0160060 | 10721144 | 10721569 | Soft_seeded |
|  | PgL0160070 | 10721719 | 10722606 | Soft_seeded |
|  | PgL0160080 | 10726964 | 10727524 | Soft_seeded |
|  | PgL0160090 | 10727974 | 10730939 | Soft_seeded |
|  | PgL0160100 | 10736469 | 10739968 | Soft_seeded |
|  | PgL0160110 | 10751231 | 10751799 | Soft_seeded |
|  | PgL0160120 | 10764014 | 10765266 | Soft_seeded |
|  | PgL0160130 | 10770460 | 10772532 | Soft_seeded |
|  | PgL0160140 | 10791442 | 10798946 | Soft_seeded |
|  | PgL0160150 | 10809170 | 10809496 | Soft_seeded |
|  | PgL0160160 | 10840850 | 10842660 | Soft_seeded |
|  | PgL0160170 | 10868882 | 10871044 | Soft_seeded |
|  | PgL0160180 | 10874166 | 10876155 | Soft_seeded |
|  | PgL0160190 | 10877622 | 10878674 | Soft_seeded |
|  | PgL0160200 | 10886892 | 10889270 | Soft_seeded |
| Chr3 | PgL0145540 | 158376 | 160991 | Soft_seeded |
|  | PgL0145550 | 181629 | 184095 | Soft_seeded |
|  | PgL0145560 | 184647 | 186282 | Soft_seeded |
|  | PgL0145570 | 198569 | 201745 | Soft_seeded |
|  | PgL0145580 | 203347 | 204150 | Soft_seeded |
|  | PgL0145590 | 204347 | 205466 | Soft_seeded |
|  | PgL0145600 | 206562 | 207853 | Soft_seeded |
|  | PgL0145610 | 211562 | 214518 | Soft_seeded |
|  | PgL0145620 | 209720 | 210001 | Soft_seeded |
|  | PgL0145630 | 222840 | 223271 | Soft_seeded |
|  | PgL0145640 | 224373 | 227532 | Soft_seeded |
|  | PgL0145650 | 230835 | 236342 | Soft_seeded |
|  | PgL0145660 | 238397 | 240258 | Soft_seeded |
|  | PgL0145670 | 250094 | 251830 | Soft_seeded |
|  | PgL0145680 | 256561 | 261582 | Soft_seeded |
|  | PgL0145690 | 266900 | 267868 | Soft_seeded |
|  | PgL0145700 | 282284 | 285874 | Soft_seeded |
|  | PgL0145710 | 290811 | 291209 | Soft_seeded |
|  | PgL0145720 | 296976 | 300218 | Soft_seeded |
|  | PgL0145730 | 303927 | 305449 | Soft_seeded |
|  | PgL0145740 | 311782 | 314854 | Soft_seeded |
|  | PgL0145750 | 317850 | 320607 | Soft_seeded |
|  | PgL0145760 | 321524 | 326092 | Soft_seeded |
|  | PgL0145770 | 327678 | 330169 | Soft_seeded |
|  | PgL0145780 | 335626 | 335847 | Soft_seeded |
|  | PgL0145790 | 335951 | 336361 | Soft_seeded |
|  | PgL0145800 | 340412 | 340636 | Soft_seeded |
|  | PgL0145810 | 341247 | 345377 | Soft_seeded |
|  | PgL0145820 | 348540 | 350997 | Soft_seeded |
|  | PgL0145830 | 353404 | 353831 | Soft_seeded |
|  | PgL0145840 | 355313 | 357217 | Soft_seeded |
|  | PgL0145850 | 358240 | 358998 | Soft_seeded |
|  | PgL0145860 | 359670 | 362691 | Soft_seeded |
|  | PgL0145870 | 364469 | 367589 | Soft_seeded |
|  | PgL0145890 | 369808 | 374150 | Soft_seeded |
| Chr3 | PgL0178290 | 34017071 | 34020174 | Soft_seeded |
|  | PgL0178300 | 34026608 | 34030987 | Soft_seeded |
|  | PgL0178310 | 34020949 | 34024341 | Soft_seeded |
|  | PgL0178320 | 34025906 | 34026202 | Soft_seeded |
|  | PgL0178330 | 34032457 | 34033926 | Soft_seeded |
|  | PgL0178340 | 34034600 | 34034962 | Soft_seeded |
|  | PgL0178350 | 34039539 | 34040339 | Soft_seeded |
|  | PgL0178360 | 34056224 | 34057156 | Soft_seeded |
|  | PgL0178370 | 34064928 | 34065839 | Soft_seeded |
|  | PgL0178380 | 34074265 | 34075170 | Soft_seeded |
|  | PgL0178390 | 34087501 | 34088172 | Soft_seeded |
|  | PgL0178400 | 34106277 | 34106783 | Soft_seeded |
|  | PgL0178410 | 34105828 | 34106208 | Soft_seeded |
|  | PgL0178420 | 34114973 | 34116576 | Soft_seeded |
|  | PgL0178430 | 34108496 | 34108867 | Soft_seeded |
|  | PgL0178440 | 34123231 | 34134865 | Soft_seeded |
|  | PgL0178450 | 34141504 | 34144182 | Soft_seeded |
|  | PgL0178460 | 34176742 | 34178835 | Soft_seeded |
|  | PgL0178470 | 34152126 | 34155580 | Soft_seeded |
|  | PgL0178480 | 34164728 | 34166532 | Soft_seeded |
|  | PgL0178490 | 34174207 | 34174632 | Soft_seeded |
|  | PgL0178500 | 34188257 | 34189696 | Soft_seeded |
|  | PgL0178510 | 34194841 | 34196280 | Soft_seeded |
|  | PgL0178520 | 34206791 | 34208488 | Soft_seeded |
|  | PgL0178530 | 34204167 | 34205579 | Soft_seeded |
|  | PgL0178540 | 34201426 | 34202865 | Soft_seeded |
| Chr3 | PgL0179680 | 35019788 | 35020574 | Soft_seeded |
|  | PgL0179690 | 35021758 | 35024613 | Soft_seeded |
|  | PgL0179700 | 35028044 | 35031593 | Soft_seeded |
|  | PgL0179710 | 35043627 | 35050720 | Soft_seeded |
|  | PgL0179720 | 35052985 | 35053272 | Soft_seeded |
|  | PgL0179730 | 35079712 | 35081073 | Soft_seeded |
|  | PgL0179740 | 35088589 | 35088864 | Soft_seeded |
|  | PgL0179750 | 35097306 | 35101753 | Soft_seeded |
|  | PgL0179760 | 35103753 | 35104166 | Soft_seeded |
|  | PgL0179770 | 35104708 | 35108085 | Soft_seeded |
|  | PgL0179780 | 35110263 | 35112913 | Soft_seeded |
|  | PgL0179790 | 35128409 | 35135327 | Soft_seeded |
|  | PgL0179800 | 35136128 | 35140818 | Soft_seeded |
|  | PgL0179810 | 35148053 | 35149384 | Soft_seeded |
|  | PgL0179820 | 35150567 | 35155807 | Soft_seeded |
|  | PgL0179830 | 35156731 | 35165025 | Soft_seeded |
| Chr4 | PgL0209030 | 15322384 | 15322857 | Soft_seeded |
|  | PgL0209040 | 15325606 | 15326625 | Soft_seeded |
|  | PgL0209050 | 15326932 | 15329797 | Soft_seeded |
|  | PgL0209060 | 15333644 | 15336163 | Soft_seeded |
|  | PgL0209070 | 15337954 | 15339313 | Soft_seeded |
|  | PgL0209080 | 15339891 | 15341368 | Soft_seeded |
|  | PgL0209090 | 15350951 | 15352124 | Soft_seeded |
|  | PgL0209100 | 15354445 | 15358405 | Soft_seeded |
|  | PgL0209110 | 15358692 | 15362535 | Soft_seeded |
|  | PgL0209120 | 15363549 | 15364495 | Soft_seeded |
|  | PgL0209130 | 15375559 | 15379169 | Soft_seeded |
|  | PgL0209140 | 15382751 | 15383497 | Soft_seeded |
|  | PgL0209150 | 15392354 | 15393237 | Soft_seeded |
|  | PgL0209160 | 15403443 | 15403772 | Soft_seeded |
|  | PgL0209170 | 15408722 | 15409729 | Soft_seeded |
|  | PgL0209180 | 15414568 | 15416057 | Soft_seeded |
|  | PgL0209190 | 15418231 | 15420584 | Soft_seeded |
|  | PgL0209200 | 15424609 | 15427607 | Soft_seeded |
|  | PgL0209210 | 15433461 | 15435393 | Soft_seeded |
|  | PgL0209220 | 15435445 | 15436911 | Soft_seeded |
|  | PgL0209230 | 15445523 | 15448580 | Soft_seeded |
|  | PgL0209240 | 15450051 | 15451320 | Soft_seeded |
|  | PgL0209250 | 15451417 | 15451809 | Soft_seeded |
|  | PgL0209260 | 15466119 | 15469853 | Soft_seeded |
|  | PgL0209270 | 15475040 | 15475642 | Soft_seeded |
|  | PgL0209280 | 15488594 | 15489085 | Soft_seeded |
|  | PgL0209290 | 15492379 | 15496062 | Soft_seeded |
|  | PgL0209300 | 15496893 | 15497849 | Soft_seeded |
| Chr5 | PgL0252220 | 11350709 | 11351068 | Soft_seeded |
|  | PgL0252230 | 11354726 | 11358312 | Soft_seeded |
|  | PgL0252240 | 11361898 | 11362764 | Soft_seeded |
|  | PgL0252250 | 11367407 | 11368090 | Soft_seeded |
|  | PgL0252260 | 11374448 | 11377298 | Soft_seeded |
|  | PgL0252270 | 11377742 | 11380423 | Soft_seeded |
|  | PgL0252280 | 11380727 | 11386377 | Soft_seeded |
|  | PgL0252290 | 11391325 | 11393246 | Soft_seeded |
|  | PgL0252300 | 11398578 | 11400545 | Soft_seeded |
|  | PgL0252310 | 11406081 | 11406962 | Soft_seeded |
|  | PgL0252320 | 11416595 | 11417205 | Soft_seeded |
|  | PgL0252330 | 11424607 | 11426872 | Soft_seeded |
|  | PgL0252340 | 11443092 | 11449120 | Soft_seeded |
|  | PgL0252350 | 11449947 | 11450258 | Soft_seeded |
|  | PgL0252360 | 11449588 | 11449887 | Soft_seeded |
|  | PgL0252370 | 11456683 | 11457960 | Soft_seeded |
|  | PgL0252380 | 11463779 | 11464465 | Soft_seeded |
|  | PgL0252390 | 11469021 | 11469703 | Soft_seeded |
|  | PgL0252400 | 11477331 | 11478017 | Soft_seeded |
|  | PgL0252410 | 11484248 | 11484818 | Soft_seeded |
|  | PgL0252420 | 11486018 | 11487292 | Soft_seeded |
|  | PgL0252430 | 11487676 | 11488148 | Soft_seeded |
|  | PgL0252440 | 11489130 | 11494501 | Soft_seeded |
|  | PgL0252450 | 11497527 | 11498021 | Soft_seeded |
|  | PgL0252460 | 11502077 | 11502442 | Soft_seeded |
|  | PgL0252470 | 11504794 | 11512333 | Soft_seeded |
|  | PgL0252480 | 11521418 | 11523389 | Soft_seeded |
|  | PgL0252490 | 11525357 | 11526165 | Soft_seeded |
|  | PgL0252500 | 11533253 | 11538856 | Soft_seeded |
| Chr5 | PgL0261810 | 23406214 | 23406501 | Soft_seeded |
|  | PgL0261820 | 23439398 | 23440029 | Soft_seeded |
|  | PgL0261830 | 23478983 | 23479348 | Soft_seeded |
|  | PgL0261840 | 23479864 | 23480166 | Soft_seeded |
|  | PgL0261850 | 23482241 | 23490317 | Soft_seeded |
|  | PgL0261860 | 23509070 | 23512037 | Soft_seeded |
|  | PgL0261870 | 23513402 | 23513974 | Soft_seeded |
|  | PgL0261880 | 23514372 | 23515778 | Soft_seeded |
|  | PgL0261890 | 23523284 | 23526757 | Soft_seeded |
|  | PgL0261900 | 23530926 | 23531198 | Soft_seeded |
|  | PgL0261910 | 23531221 | 23531472 | Soft_seeded |
|  | PgL0261920 | 23532816 | 23533136 | Soft_seeded |
|  | PgL0261930 | 23534295 | 23536021 | Soft_seeded |
|  | PgL0261940 | 23545210 | 23547731 | Soft_seeded |
|  | PgL0261950 | 23552372 | 23554849 | Soft_seeded |
| Chr6 | PgL0293500 | 23449455 | 23451735 | Soft_seeded |
|  | PgL0293510 | 23455923 | 23459064 | Soft_seeded |
|  | PgL0293520 | 23460499 | 23461534 | Soft_seeded |
|  | PgL0293530 | 23464969 | 23466404 | Soft_seeded |
|  | PgL0293540 | 23493309 | 23496126 | Soft_seeded |
|  | PgL0293550 | 23497518 | 23498108 | Soft_seeded |
|  | PgL0293560 | 23499752 | 23501988 | Soft_seeded |
|  | PgL0293570 | 23506751 | 23510494 | Soft_seeded |
|  | PgL0293580 | 23511184 | 23513019 | Soft_seeded |
|  | PgL0293590 | 23517697 | 23519994 | Soft_seeded |
|  | PgL0293600 | 23540550 | 23545955 | Soft_seeded |
|  | PgL0293610 | 23547437 | 23547769 | Soft_seeded |
|  | PgL0293620 | 23550254 | 23557209 | Soft_seeded |
| Chr8 | PgL0018900 | 13317924 | 13322869 | Soft_seeded |
|  | PgL0018910 | 13329148 | 13329669 | Soft_seeded |
|  | PgL0018920 | 13340842 | 13344798 | Soft_seeded |
|  | PgL0018930 | 13346305 | 13347100 | Soft_seeded |
|  | PgL0018940 | 13348459 | 13349689 | Soft_seeded |
|  | PgL0018950 | 13369534 | 13370010 | Soft_seeded |
|  | PgL0018960 | 13370048 | 13373486 | Soft_seeded |
|  | PgL0018970 | 13378510 | 13379178 | Soft_seeded |
|  | PgL0018980 | 13390987 | 13391710 | Soft_seeded |
|  | PgL0018990 | 13394846 | 13403165 | Soft_seeded |
| Chr8 | PgL0028460 | 24160673 | 24161095 | Soft_seeded |
|  | PgL0028470 | 24161641 | 24162066 | Soft_seeded |
|  | PgL0028480 | 24168308 | 24168598 | Soft_seeded |
|  | PgL0028490 | 24173138 | 24173425 | Soft_seeded |
|  | PgL0028500 | 24175643 | 24176329 | Soft_seeded |
|  | PgL0028510 | 24190306 | 24194059 | Soft_seeded |
|  | PgL0028520 | 24198519 | 24203318 | Soft_seeded |
|  | PgL0028530 | 24206853 | 24207155 | Soft_seeded |
|  | PgL0028540 | 24210663 | 24212542 | Soft_seeded |
|  | PgL0028550 | 24216241 | 24217036 | Soft_seeded |
|  | PgL0028560 | 24215534 | 24216190 | Soft_seeded |
|  | PgL0028570 | 24222105 | 24225838 | Soft_seeded |
|  | PgL0028580 | 24233021 | 24237761 | Soft_seeded |
|  | PgL0028590 | 24242163 | 24244692 | Soft_seeded |
|  | PgL0028600 | 24266756 | 24267679 | Soft_seeded |
|  | PgL0028610 | 24276646 | 24276981 | Soft_seeded |
|  | PgL0028620 | 24277222 | 24280599 | Soft_seeded |
| Chr1 | PgL0110300 | 10047063 | 10050291 | Hard_seeded |
|  | PgL0110310 | 10058434 | 10074328 | Hard_seeded |
|  | PgL0110320 | 10076293 | 10077084 | Hard_seeded |
|  | PgL0110330 | 10079017 | 10079769 | Hard_seeded |
|  | PgL0110340 | 10082788 | 10087255 | Hard_seeded |
|  | PgL0110350 | 10090084 | 10090742 | Hard_seeded |
|  | PgL0110360 | 10091296 | 10094259 | Hard_seeded |
|  | PgL0110370 | 10095698 | 10098169 | Hard_seeded |
|  | PgL0110380 | 10101741 | 10103255 | Hard_seeded |
|  | PgL0110390 | 10104696 | 10105397 | Hard_seeded |
|  | PgL0110400 | 10110768 | 10111299 | Hard_seeded |
|  | PgL0110410 | 10118020 | 10122131 | Hard_seeded |
|  | PgL0110420 | 10122931 | 10125777 | Hard_seeded |
|  | PgL0110430 | 10129731 | 10131236 | Hard_seeded |
|  | PgL0110440 | 10136668 | 10141002 | Hard_seeded |
|  | PgL0110450 | 10148017 | 10150153 | Hard_seeded |
|  | PgL0110460 | 10158502 | 10165693 | Hard_seeded |
|  | PgL0110470 | 10166097 | 10169200 | Hard_seeded |
|  | PgL0110480 | 10170763 | 10178694 | Hard_seeded |
|  | PgL0110490 | 10180033 | 10180278 | Hard_seeded |
|  | PgL0110500 | 10185752 | 10185985 | Hard_seeded |
| Chr2 | PgL0044500 | 2289455 | 2290959 | Hard_seeded |
|  | PgL0044510 | 2296445 | 2298367 | Hard_seeded |
|  | PgL0044520 | 2301549 | 2305016 | Hard_seeded |
|  | PgL0044530 | 2325140 | 2328747 | Hard_seeded |
|  | PgL0044540 | 2333434 | 2334069 | Hard_seeded |
|  | PgL0044550 | 2334753 | 2335908 | Hard_seeded |
|  | PgL0044560 | 2335968 | 2336571 | Hard_seeded |
|  | PgL0044570 | 2350391 | 2353634 | Hard_seeded |
|  | PgL0044580 | 2354750 | 2358874 | Hard_seeded |
|  | PgL0044590 | 2359887 | 2360129 | Hard_seeded |
|  | PgL0044600 | 2362871 | 2364748 | Hard_seeded |
|  | PgL0044610 | 2365618 | 2377149 | Hard_seeded |
|  | PgL0044620 | 2383360 | 2384240 | Hard_seeded |
|  | PgL0044630 | 2391202 | 2391678 | Hard_seeded |
|  | PgL0044640 | 2401332 | 2404194 | Hard_seeded |
|  | PgL0044650 | 2405095 | 2409715 | Hard_seeded |
|  | PgL0044660 | 2438422 | 2438745 | Hard_seeded |
|  | PgL0044670 | 2449273 | 2455078 | Hard_seeded |
|  | PgL0044680 | 2455320 | 2455565 | Hard_seeded |
|  | PgL0044690 | 2456528 | 2458587 | Hard_seeded |
|  | PgL0044700 | 2459856 | 2460782 | Hard_seeded |
|  | PgL0044710 | 2460977 | 2461360 | Hard_seeded |
|  | PgL0044720 | 2461765 | 2463523 | Hard_seeded |
|  | PgL0044730 | 2465815 | 2469967 | Hard_seeded |
|  | PgL0044740 | 2470387 | 2471190 | Hard_seeded |
|  | PgL0044750 | 2471317 | 2471634 | Hard_seeded |
|  | PgL0044760 | 2472663 | 2473896 | Hard_seeded |
|  | PgL0044770 | 2475103 | 2477066 | Hard_seeded |
|  | PgL0044780 | 2477975 | 2480474 | Hard_seeded |
| Chr4 | PgL0218380 | 27637551 | 27637985 | Hard_seeded |
|  | PgL0218390 | 27648787 | 27648948 | Hard_seeded |
|  | PgL0218400 | 27652233 | 27653322 | Hard_seeded |
|  | PgL0218410 | 27661293 | 27662030 | Hard_seeded |
|  | PgL0218420 | 27662336 | 27662647 | Hard_seeded |
|  | PgL0218430 | 27678903 | 27679784 | Hard_seeded |
|  | PgL0218440 | 27682307 | 27691555 | Hard_seeded |
|  | PgL0218450 | 27709205 | 27711778 | Hard_seeded |
|  | PgL0218460 | 27723913 | 27724587 | Hard_seeded |
| Chr5 | PgL0266300 | 28048958 | 28050101 | Hard_seeded |
|  | PgL0266310 | 28054409 | 28054642 | Hard_seeded |
|  | PgL0266320 | 28062600 | 28063058 | Hard_seeded |
|  | PgL0266330 | 28075854 | 28077392 | Hard_seeded |
|  | PgL0266340 | 28086986 | 28091865 | Hard_seeded |
|  | PgL0266350 | 28093788 | 28094414 | Hard_seeded |
|  | PgL0266360 | 28102395 | 28103613 | Hard_seeded |
|  | PgL0266370 | 28106014 | 28108412 | Hard_seeded |
|  | PgL0266380 | 28118808 | 28121102 | Hard_seeded |
|  | PgL0266390 | 28124710 | 28125648 | Hard_seeded |
|  | PgL0266400 | 28141626 | 28143168 | Hard_seeded |
|  | PgL0266410 | 28144771 | 28147134 | Hard_seeded |
|  | PgL0266420 | 28149243 | 28149733 | Hard_seeded |
| Chr7 | PgL0312250 | 7055648 | 7057029 | Hard_seeded |
|  | PgL0312260 | 7064939 | 7069243 | Hard_seeded |
|  | PgL0312270 | 7070597 | 7073842 | Hard_seeded |
|  | PgL0312280 | 7074525 | 7075166 | Hard_seeded |
|  | PgL0312290 | 7087474 | 7088655 | Hard_seeded |
|  | PgL0312300 | 7113245 | 7118795 | Hard_seeded |
|  | PgL0312310 | 7121370 | 7121708 | Hard_seeded |
|  | PgL0312320 | 7143660 | 7146910 | Hard_seeded |
| Chr7 | PgL0312380 | 7188392 | 7188844 | Hard_seeded |
|  | PgL0312390 | 7188952 | 7191486 | Hard_seeded |
|  | PgL0312400 | 7194719 | 7195116 | Hard_seeded |
|  | PgL0312410 | 7195195 | 7196375 | Hard_seeded |
|  | PgL0312420 | 7221837 | 7222877 | Hard_seeded |
|  | PgL0312430 | 7237323 | 7239869 | Hard_seeded |
|  | PgL0312440 | 7241063 | 7241893 | Hard_seeded |
|  | PgL0312450 | 7242340 | 7242882 | Hard_seeded |
|  | PgL0312460 | 7244388 | 7244819 | Hard_seeded |
|  | PgL0312470 | 7247507 | 7248127 | Hard_seeded |
|  | PgL0312480 | 7252135 | 7252416 | Hard_seeded |
|  | PgL0312490 | 7252467 | 7256439 | Hard_seeded |
|  | PgL0312500 | 7256951 | 7258661 | Hard_seeded |
|  | PgL0312510 | 7260325 | 7262110 | Hard_seeded |
|  | PgL0312520 | 7272435 | 7274894 | Hard_seeded |
|  | PgL0312530 | 7276430 | 7278140 | Hard_seeded |
|  | PgL0312540 | 7280402 | 7282656 | Hard_seeded |
|  | PgL0312550 | 7295223 | 7295806 | Hard_seeded |
|  | PgL0312560 | 7298878 | 7299432 | Hard_seeded |
|  | PgL0312570 | 7309858 | 7310385 | Hard_seeded |
|  | PgL0312580 | 7315128 | 7318152 | Hard_seeded |
|  | PgL0312590 | 7320270 | 7321775 | Hard_seeded |
|  | PgL0312600 | 7327375 | 7331804 | Hard_seeded |
|  | PgL0312610 | 7335488 | 7336729 | Hard_seeded |
|  | PgL0312620 | 7336793 | 7337092 | Hard_seeded |
|  | PgL0312630 | 7338277 | 7342752 | Hard_seeded |
| Chr7 | PgL0313210 | 7889691 | 7890177 | Hard_seeded |
|  | PgL0313220 | 7891117 | 7893021 | Hard_seeded |
|  | PgL0313230 | 7894124 | 7895354 | Hard_seeded |
|  | PgL0313240 | 7899816 | 7900347 | Hard_seeded |
|  | PgL0313250 | 7909709 | 7910639 | Hard_seeded |
|  | PgL0313260 | 7910785 | 7912845 | Hard_seeded |
|  | PgL0313270 | 7914366 | 7915355 | Hard_seeded |
|  | PgL0313280 | 7917598 | 7919815 | Hard_seeded |
|  | PgL0313290 | 7925886 | 7927260 | Hard_seeded |
|  | PgL0313300 | 7930195 | 7932197 | Hard_seeded |
|  | PgL0313310 | 7942263 | 7944161 | Hard_seeded |
|  | PgL0313320 | 7944870 | 7951017 | Hard_seeded |
|  | PgL0313330 | 7962528 | 7977544 | Hard_seeded |
|  | PgL0313340 | 7984179 | 7988407 | Hard_seeded |
|  | PgL0313350 | 7998637 | 7999573 | Hard_seeded |
|  | PgL0313360 | 8003434 | 8003819 | Hard_seeded |
|  | PgL0313370 | 8007749 | 8010215 | Hard_seeded |
|  | PgL0313380 | 8013296 | 8014442 | Hard_seeded |
|  | PgL0313390 | 8015717 | 8015965 | Hard_seeded |
|  | PgL0313400 | 8028789 | 8035816 | Hard_seeded |
|  | PgL0313410 | 8042691 | 8043095 | Hard_seeded |
|  | PgL0313420 | 8045428 | 8047678 | Hard_seeded |
|  | PgL0313430 | 8051354 | 8051602 | Hard_seeded |
|  | PgL0313450 | 8093096 | 8094490 | Hard_seeded |
|  | PgL0313460 | 8091609 | 8092600 | Hard_seeded |
|  | PgL0313470 | 8123337 | 8123879 | Hard_seeded |
| Chr7 | PgL0313510 | 8168468 | 8172206 | Hard_seeded |
|  | PgL0313520 | 8178479 | 8184030 | Hard_seeded |
|  | PgL0313530 | 8191308 | 8193566 | Hard_seeded |
|  | PgL0313540 | 8211375 | 8211704 | Hard_seeded |
|  | PgL0313550 | 8213823 | 8226101 | Hard_seeded |
|  | PgL0313560 | 8229115 | 8229744 | Hard_seeded |
|  | PgL0313570 | 8231671 | 8235025 | Hard_seeded |
|  | PgL0313580 | 8236042 | 8243979 | Hard_seeded |
|  | PgL0313590 | 8244232 | 8250579 | Hard_seeded |
|  | PgL0313600 | 8252153 | 8252765 | Hard_seeded |
|  | PgL0313610 | 8259628 | 8264717 | Hard_seeded |
|  | PgL0313620 | 8268096 | 8270440 | Hard_seeded |
|  | PgL0313630 | 8271012 | 8272019 | Hard_seeded |
|  | PgL0313640 | 8291243 | 8297189 | Hard_seeded |
|  | PgL0313650 | 8299885 | 8301762 | Hard_seeded |
|  | PgL0313660 | 8304319 | 8305687 | Hard_seeded |
|  | PgL0313670 | 8316218 | 8321007 | Hard_seeded |
|  | PgL0313680 | 8321052 | 8321554 | Hard_seeded |
|  | PgL0313690 | 8325307 | 8330091 | Hard_seeded |
|  | PgL0313700 | 8332384 | 8335454 | Hard_seeded |
|  | PgL0313710 | 8336266 | 8341663 | Hard_seeded |
|  | PgL0313720 | 8345784 | 8346161 | Hard_seeded |
|  | PgL0313730 | 8361521 | 8363902 | Hard_seeded |
|  | PgL0313740 | 8382834 | 8383820 | Hard_seeded |
|  | PgL0313750 | 8385194 | 8397356 | Hard_seeded |
|  | PgL0313760 | 8399675 | 8401176 | Hard_seeded |
|  | PgL0313770 | 8397381 | 8399642 | Hard_seeded |
|  | PgL0313780 | 8406181 | 8410347 | Hard_seeded |
|  | PgL0313790 | 8435016 | 8438843 | Hard_seeded |
|  | PgL0313800 | 8440874 | 8446539 | Hard_seeded |
| Chr7 | PgL0314270 | 8898981 | 8901563 | Hard_seeded |
|  | PgL0314280 | 8905120 | 8907666 | Hard_seeded |
|  | PgL0314290 | 8911488 | 8914066 | Hard_seeded |
|  | PgL0314300 | 8917873 | 8920452 | Hard_seeded |
|  | PgL0314310 | 8924222 | 8925385 | Hard_seeded |
|  | PgL0314320 | 8929416 | 8930381 | Hard_seeded |
|  | PgL0314330 | 8931908 | 8935056 | Hard_seeded |
|  | PgL0314340 | 8937852 | 8939867 | Hard_seeded |
|  | PgL0314350 | 8941968 | 8942273 | Hard_seeded |
|  | PgL0314360 | 8944562 | 8945470 | Hard_seeded |
|  | PgL0314370 | 8946682 | 8951258 | Hard_seeded |
|  | PgL0314380 | 8951496 | 8955219 | Hard_seeded |
|  | PgL0314390 | 8959750 | 8960652 | Hard_seeded |
|  | PgL0314400 | 8969149 | 8973352 | Hard_seeded |
|  | PgL0314410 | 8974036 | 8977763 | Hard_seeded |
|  | PgL0314420 | 8991616 | 8994745 | Hard_seeded |
|  | PgL0314430 | 8997265 | 8997711 | Hard_seeded |
|  | PgL0314340 | 8937852 | 8939867 | Hard_seeded |
|  | PgL0314350 | 8941968 | 8942273 | Hard_seeded |
|  | PgL0314360 | 8944562 | 8945470 | Hard_seeded |
|  | PgL0314370 | 8946682 | 8951258 | Hard_seeded |
|  | PgL0314380 | 8951496 | 8955219 | Hard_seeded |
|  | PgL0314390 | 8959750 | 8960652 | Hard_seeded |
|  | PgL0314400 | 8969149 | 8973352 | Hard_seeded |
|  | PgL0314410 | 8974036 | 8977763 | Hard_seeded |
|  | PgL0314420 | 8991616 | 8994745 | Hard_seeded |
|  | PgL0314430 | 8997265 | 8997711 | Hard_seeded |
|  | PgL0314440 | 9007161 | 9007535 | Hard_seeded |
|  | PgL0314450 | 9007985 | 9009787 | Hard_seeded |
|  | PgL0314460 | 9023238 | 9023516 | Hard_seeded |
|  | PgL0314470 | 9038322 | 9038546 | Hard_seeded |
|  | PgL0314480 | 9042716 | 9045133 | Hard_seeded |
|  | PgL0314490 | 9048299 | 9048622 | Hard_seeded |
|  | PgL0314500 | 9057802 | 9060487 | Hard_seeded |
|  | PgL0314510 | 9083019 | 9085197 | Hard_seeded |
|  | PgL0314520 | 9100745 | 9103566 | Hard_seeded |
|  | PgL0314530 | 9106037 | 9109356 | Hard_seeded |
|  | PgL0314540 | 9116542 | 9116937 | Hard_seeded |
|  | PgL0314550 | 9126808 | 9129276 | Hard_seeded |
| Chr7 | PgL0314630 | 9242763 | 9244946 | Hard_seeded |
|  | PgL0314640 | 9248408 | 9249365 | Hard_seeded |
|  | PgL0314650 | 9254032 | 9256398 | Hard_seeded |
|  | PgL0314660 | 9260844 | 9262628 | Hard_seeded |
|  | PgL0314670 | 9263738 | 9271190 | Hard_seeded |
|  | PgL0314680 | 9279957 | 9281243 | Hard_seeded |
|  | PgL0314690 | 9310317 | 9310850 | Hard_seeded |
|  | PgL0314700 | 9319060 | 9319401 | Hard_seeded |
|  | PgL0314710 | 9387871 | 9388870 | Hard_seeded |
|  | PgL0314720 | 9419930 | 9421750 | Hard_seeded |
|  | PgL0314730 | 9447780 | 9448686 | Hard_seeded |
|  | PgL0314740 | 9455964 | 9457676 | Hard_seeded |
|  | PgL0314750 | 9470092 | 9473222 | Hard_seeded |
|  | PgL0314760 | 9476577 | 9477065 | Hard_seeded |
|  | PgL0314770 | 9481668 | 9482006 | Hard_seeded |
|  | PgL0314780 | 9482604 | 9482987 | Hard_seeded |
|  | PgL0314790 | 9492109 | 9492525 | Hard_seeded |
|  | PgL0314800 | 9493686 | 9494603 | Hard_seeded |
|  | PgL0314810 | 9506542 | 9510516 | Hard_seeded |
|  | PgL0314820 | 9513931 | 9518472 | Hard_seeded |
|  | PgL0314830 | 9518890 | 9519285 | Hard_seeded |
|  | PgL0314840 | 9528354 | 9529826 | Hard_seeded |
|  | PgL0314850 | 9530333 | 9531328 | Hard_seeded |
|  | PgL0314860 | 9572487 | 9572783 | Hard_seeded |
|  | PgL0314870 | 9580940 | 9581395 | Hard_seeded |
|  | PgL0314880 | 9590157 | 9590579 | Hard_seeded |
|  | PgL0314890 | 9597763 | 9598038 | Hard_seeded |
|  | PgL0314900 | 9603757 | 9606976 | Hard_seeded |
|  | PgL0314910 | 9608115 | 9610819 | Hard_seeded |
|  | PgL0314920 | 9613222 | 9615411 | Hard_seeded |
|  | PgL0314930 | 9618332 | 9618763 | Hard_seeded |
|  | PgL0314940 | 9642175 | 9643620 | Hard_seeded |
|  | PgL0314950 | 9651844 | 9661999 | Hard_seeded |
|  | PgL0314960 | 9663921 | 9664223 | Hard_seeded |
|  | PgL0314970 | 9687194 | 9688681 | Hard_seeded |
|  | PgL0314980 | 9690558 | 9691306 | Hard_seeded |
|  | PgL0314990 | 9712912 | 9717757 | Hard_seeded |
|  | PgL0315000 | 9718394 | 9719275 | Hard_seeded |
|  | PgL0315010 | 9720544 | 9723891 | Hard_seeded |
|  | PgL0315020 | 9749696 | 9749986 | Hard_seeded |
|  | PgL0315030 | 9768889 | 9772561 | Hard_seeded |
|  | PgL0315040 | 9776590 | 9777778 | Hard_seeded |
|  | PgL0315050 | 9786413 | 9789345 | Hard_seeded |
|  | PgL0315060 | 9807105 | 9810616 | Hard_seeded |
|  | PgL0315070 | 9836291 | 9843178 | Hard_seeded |
|  | PgL0315080 | 9832114 | 9832769 | Hard_seeded |
|  | PgL0315090 | 9843464 | 9843860 | Hard_seeded |
|  | PgL0315100 | 9901605 | 9901922 | Hard_seeded |
